# Recent Advances of Microcapsule‐based Intelligent Coatings: From Bioinspired Designs, Material Selections, to Potential Applications

**DOI:** 10.1002/advs.202506557

**Published:** 2025-07-28

**Authors:** Yue Zhao, Xiaopeng Cheng, Ran Zhao, Xipeng Li, Jingxin Meng, Shutao Wang

**Affiliations:** ^1^ Shandong Laboratory of Aluminum Advanced Manufacturing in Binzhou Binzhou Institute of Technology Binzhou 256606 P. R. China; ^2^ Laboratory of Bio‐inspired Smart Interface Science Technical Institute of Physics and Chemistry, Chinese Academy of Sciences Beijing 100190 P. R. China; ^3^ University of Chinese Academy of Sciences Beijing 100049 P. R. China

**Keywords:** bioinspired designs, intelligent coatings, microcapsules, stimulus responses

## Abstract

Coating technology is widely used in diverse fields such as energy, healthcare, and aerospace because of the advantages of simple fabrication, low cost, stability, and customized functions. However, conventional coatings fall short in meeting the emerging demands due to their single functionality and non‐responsiveness. Inspired by natural prototypes, microcapsule‐based intelligent coatings (MICs) that exhibit remarkable responsiveness to external stimuli have been developed and used in various fields such as corrosion protection and anti‐fouling. Comprehensively understanding the responsive mechanisms and optimization strategies of MICs can maximize their adaptability and flexibility, which are highly desired for their precise preparation and actual applications. Therefore, this review focuses on recent progress of MICs, varying from bioinspired designs, material selections, stimulus responses, and diverse applications, providing more inspiration to design advanced MICs. First, biological prototypes used in the fabrication of MICs are briefly introduced. Then, the material selections and stimulus responses of MICs, as well as their advantages and disadvantages are emphasized. Later, applications are focused according to their functions, including anti‐corrosion, anti‐fouling, self‐lubricating, and temperature regulation. Finally, the scientific challenges and prospects in designing MICs, focusing on the requirements of developing new structures using machine learning and achieving new applications, are highlighted.

## Graphical abstract

Microcapsule‐based intelligent coatings (MICs) have shown great potential in the fields of biomedicine, smart buildings, marine protection, and aerospace. However, they currently face challenges in the integration of coating functions and applications in complex systems. This review summarizes the recent progress of MICs in material design, stimulus response, and application fields. In addition, the future challenges development of MICs are reviewed.

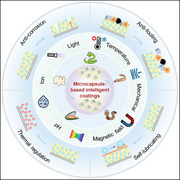

## 1 Introduction

Coating technology is an effective way to modify surface properties to satisfy specific performance requirements such as corrosion protection, lubrication, and anti‐fouling.^[^ ^1^ ^]^ Due to its advantages of simple fabrication, low cost, stability, and customizable functions, coating technology has been widely studied and used in diverse fields such as energy, life health, and aerospace.^[^ ^2^ ^]^ However, with the increasing complexity of service environments and actual requirements, traditional coatings cannot meet the emerging demands of people due to their single functionality, non‐intelligent responsiveness, and short lifespan. To address these limitations, advanced coatings incorporating microcapsules, crystalline/mineral additives, and compatibilizers have been gradually developed.^[^ ^3^ ^]^ Among them, coatings containing microcapsules, namely microcapsules‐based intelligent coatings (MICs) that are inspired by various natural prototypes, can respond in a controlled manner to the external environment and achieve desirable properties.^[^ ^4^ ^]^ The versatility, stimulus responsiveness, and flexibility of MICs exhibit greater potential for both commercial and scientific aspects compared with traditional coatings. For example, anti‐microbial door handle coatings incorporated with iodine‐loaded UiO‐66 microcapsules can efficiently prevent cross‐contamination against various bacterial species, while those unloaded coatings cannot prevent cross‐contamination, and significant bacterial growth (>200 CFU) could be found on the unloaded coatings.^[^ ^5^ ^]^ Extensive research on MICs has been carried out across diverse fields such as self‐healing,^[^ ^6^ ^]^ corrosion protection,^[^ ^7^ ^]^ and anti‐fouling.^[^ ^8^ ^]^

**Figure 1:**
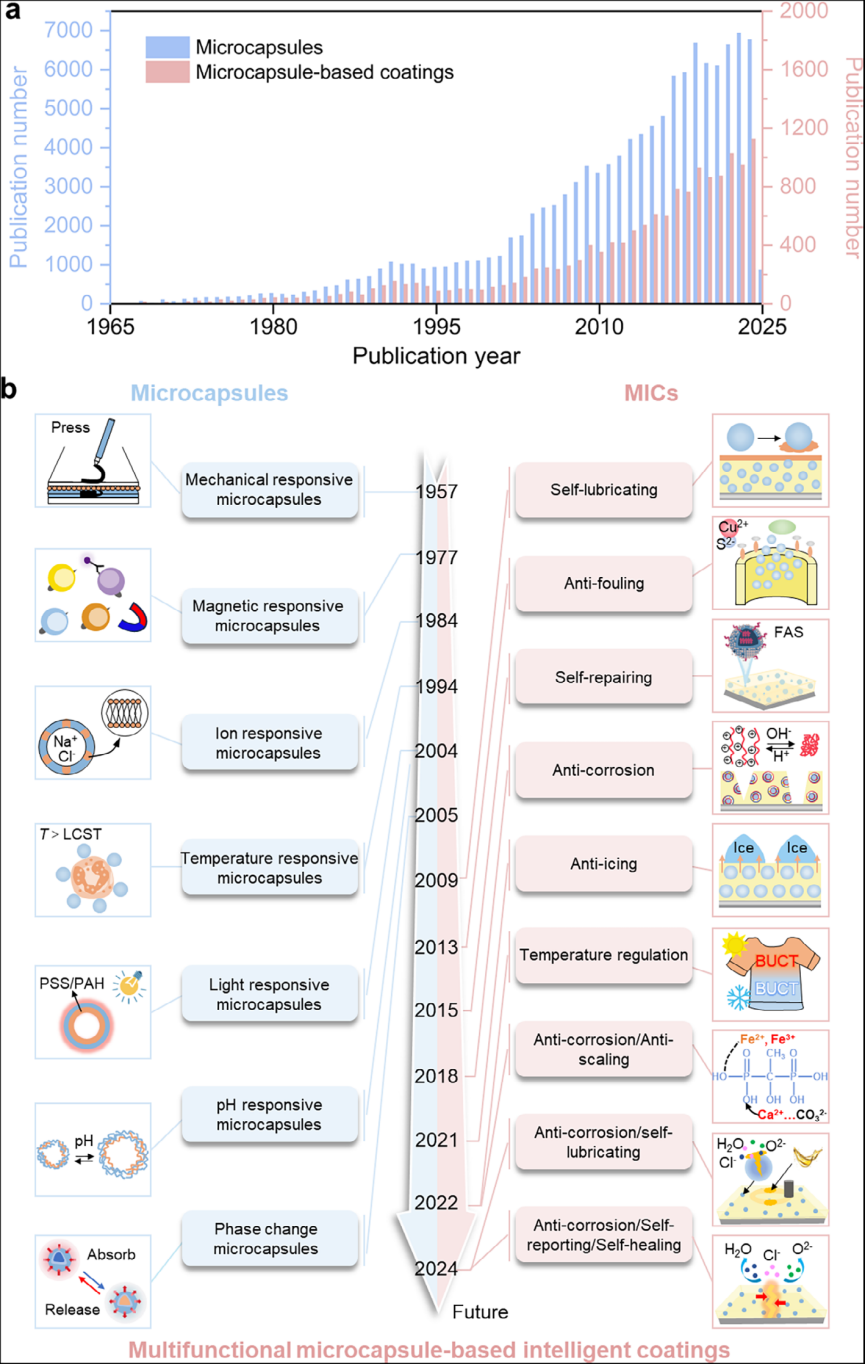
a) The publication records of microcapsules and microcapsule‐based coatings in Web of Science as on 27th March 2025. b) A brief chronology of representative progress in stimulus responsive microcapsules and MICs. Mechanical responsive microcapsules. Reproduced with permission.^[^ ^11^ ^]^ Copyright 1998, American Chemical Society. Magnetic responsive microcapsules. Reproduced with permission.^[^ ^12^ ^]^ Copyright 1977, Nature. Ion‐responsive microcapsules. Reproduced with permission.^[^ ^13^ ^]^ Copyright 1984, American Chemical Society. Temperature‐responsive microcapsules. Reproduced with permission.^[^ ^14^ ^]^ Copyright 1994, Wiley‐VCH. Light and pH‐responsive microcapsules. Reproduced with permission.^[^ ^15^, ^16^ ^]^ Copyright 2004, Wiley‐VCH. Anti‐fouling coating. Reproduced with permission.^[18f]^ Copyright 2013, Wiley‐VCH. Self‐repairing coating. Reproduced with permission.^[18a]^ Copyright 2015, Wiley‐VCH. Anti‐corrosion coating. Reproduced with permission.^[18c]^ Copyright 2018, Elsevier Ltd. Anti‐icing coating. Reproduced with permission.^[18e]^ Copyright 2021, Elsevier Ltd. Thermal regulation textile. Reproduced with permission.^[18d]^ Copyright 2022, Elsevier Ltd. Anti‐corrosion/anti‐scaling coatings. Reproduced with permission.^[19b]^ Copyright 2022, Elsevier Ltd. Anti‐corrosion/self‐lubricating coatings. Reproduced with permission.^[19a]^ Copyright 2024, American Chemical Society. Anti‐corrosion/self‐reporting/self‐healing coatings. Reproduced with permission.^[19c]^ Copyright 2024, American Chemical Society.

Generally, MICs consist of two primary components: the intelligent microcapsules and the coating matrixes. The intelligent microcapsules can release loadings in response to external stimulus, so as to achieve an intelligent response of MICs. The coating matrixes serve as a protective barrier to isolate the substrate from external media.^[^ ^9^ ^]^ To meet the urgent needs of complex service environments, MICs with diverse functions have been designed and developed by adjusting both the intelligent microcapsules and coating matrixes.^[^ ^10^ ^]^ Various preparation methods such as spraying, scraping, spinning, brushing, and dipping, have been employed for applying MICs onto substrates. In **Table** **1**, we introduce these preparation methods as well as their advantages and disadvantages. **Figure** **1**a presents a summary of the remarkable advances in MICs. Intelligent microcapsules triggered by diverse stimulus responses, including mechanical,^[^ ^11^ ^]^ magnetic,^[^ ^12^ ^]^ ion,^[^ ^13^ ^]^ temperature,^[^ ^14^ ^]^ light,^[^ ^15^ ^]^ and pH,^[^ ^16^ ^]^ have been successively developed since 1957 (the left of Figure 1b) through adjustments to shell and core materials of microcapsules. Phase change material‐based microcapsules (PCMCs) were prepared in 2005.^[^ ^17^ ^]^ These intelligent microcapsules can be incorporated into coating matrixes to prepare novel and functional MICs, which have been widely used in various fields. Since 2009, MICs with varying stimulus‐responsive functions, including self‐lubricating, anti‐fouling, self‐repairing, anti‐corrosion, anti‐icing, and thermal regulation, have been intensively studied by researchers.^[^ ^18^ ^]^ Furthermore, multi‐functional MICs can also be prepared because of the high flexibility and modularization of microcapsules. For example, MICs with combined functions such as anti‐corrosion/anti‐scaling, anti‐corrosion/self‐lubricating, and anti‐corrosion/self‐reporting/self‐healing have been gradually developed (the right of Figure 1b).^[^ ^19^ ^]^ These studies have fully demonstrated the great application prospects and potential of MICs.

**Table 1:** Summary of the preparation methods of MICs, and their advantages and disadvantages.

Methods	Advantages	Disadvantages	Influence factors	Refs.
Spraying	Fast speed; Good uniformity	Waste of paint; Pollute the environment	Distance between the sample and the airbrush; Pressure; Duration of the spray	[20]
Scraping	Simple operation; Good uniformity	Low efficiency	Solution concentration; Blade gap; Blade‐coating speed	[19, 20, 21]
Spinning	Thickness control; Excellent uniformity	Low utilization rate; Thick coatings are difficult to prepare	Viscosity; Drying rate; Rotation speed	[20, 22]
Brushing	Simple operation; Flexible and controllable thickness	Low efficiency; Uneven thickness	/	[23]
Dipping	Simple process; High production efficiency	Long drying time; Environmental pollution	pH; Solution concentration	[18, 20, 24]

**Figure 2:**
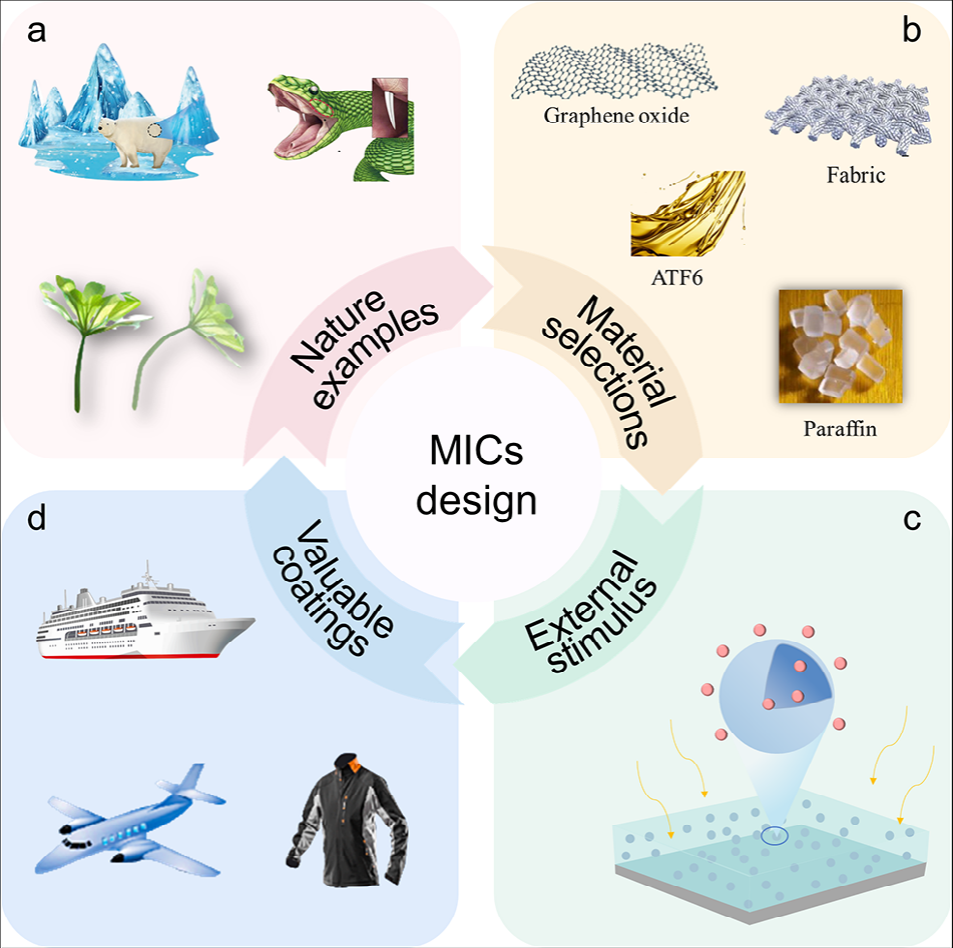
Themes covered in this review on advanced MICs design. a) Nature examples: structural features and underlying principles. Reproduced with permission.^[^ ^27^ ^]^ Copyright 2022, Wiley‐VCH. Copyright 2019, American Association for the Advancement of Science. Copyright 2025, Wiley‐VCH. b) Material selections: the selection criterion of shell, core, and coating materials. Reproduced with permission.^[^ ^28^ ^]^ Copyright 2021, Royal Society of Chemistry. Copyright 2025, Elsevier Ltd. Copyright 2020, Elsevier Ltd. Copyright 2022, American Chemical Society. c) External stimulus: single and multiple stimulus responses. d) Valuable coatings: practical applications of MICs with anti‐corrosion/anti‐fouling/self‐lubricating/temperature regulation functions. Reproduced with permission.^[^ ^8^, ^29^ ^]^ Copyright 2022, Elsevier Ltd. Copyright 2024, Elsevier Ltd.

Numerous reviews of MICs have been published to promote the development of MICs. For example, Yang's group reviewed the advances in micro/nanocontainer‐based intelligent coatings from the aspect of classification and synthesis, stimulus response, and applications of micro/nanocontainer‐based intelligent coatings.^[^ ^6b^ ^]^ Zhang and his coworkers summarized the advantages and limitations associated with common autonomous and non‐autonomous self‐healing mechanisms in protective organic coatings used for anti‐corrosion purposes.^[^ ^25^ ^]^ Zhao's group reviewed responsive self‐healing anti‐corrosion coatings from mechanism to stimuli‐response, including single response and multiple responses.^[^ ^26^ ^]^ Zehra and his colleagues conducted an analysis of reported preparation methods, triggers required for smart response, and emerging trends for smart functional coatings.^[^ ^7^ ^]^ However, previous reviews mainly focused on the application of microcapsule‐based intelligent coatings in the field of self‐healing and anti‐corrosion. Few of these studies have comprehensively integrated and analyzed the response mechanisms and optimization strategies of MICs, which are highly desired for their precise preparation and actual applications. In practical applications, the design of a reasonable intelligent response mechanism and the realization of a variety of functional synergies can maximize the adaptability and flexibility of the coating and improve the overall performance of the coating. For example, by filling the microcapsules of the MICs with scale inhibitors and corrosion inhibitors, the anti‐corrosion and anti‐scaling of the metal surface can be achieved simultaneously, which is very important for improving the service life of metal materials.^[^ ^19b^ ^]^ This review mainly focuses on the recent progress of MICs varying from four aspects of the technology: bioinspired designs, material selections, stimulus response, and diverse applications, providing us more insights for selectively designing advanced MICs (**Figure** **2**). First, we briefly introduce biological prototypes used in the design and fabrication of microcapsule‐based intelligent coatings. Then, we emphasize the material selection and the types of stimulus‐response in microcapsule‐based intelligent coatings. The advantages and disadvantages of MICs are also analyzed in detail. Later, we focus on the applications of microcapsule‐based intelligent coatings in various fields such as anti‐corrosion, anti‐fouling, self‐lubricating, and temperature regulation. Finally, we highlight the scientific challenges and promising prospects of MICs, particularly focusing on the requirements for developing new structures and achieving new applications.

## 2 Nature Examples

**Figure 3:**
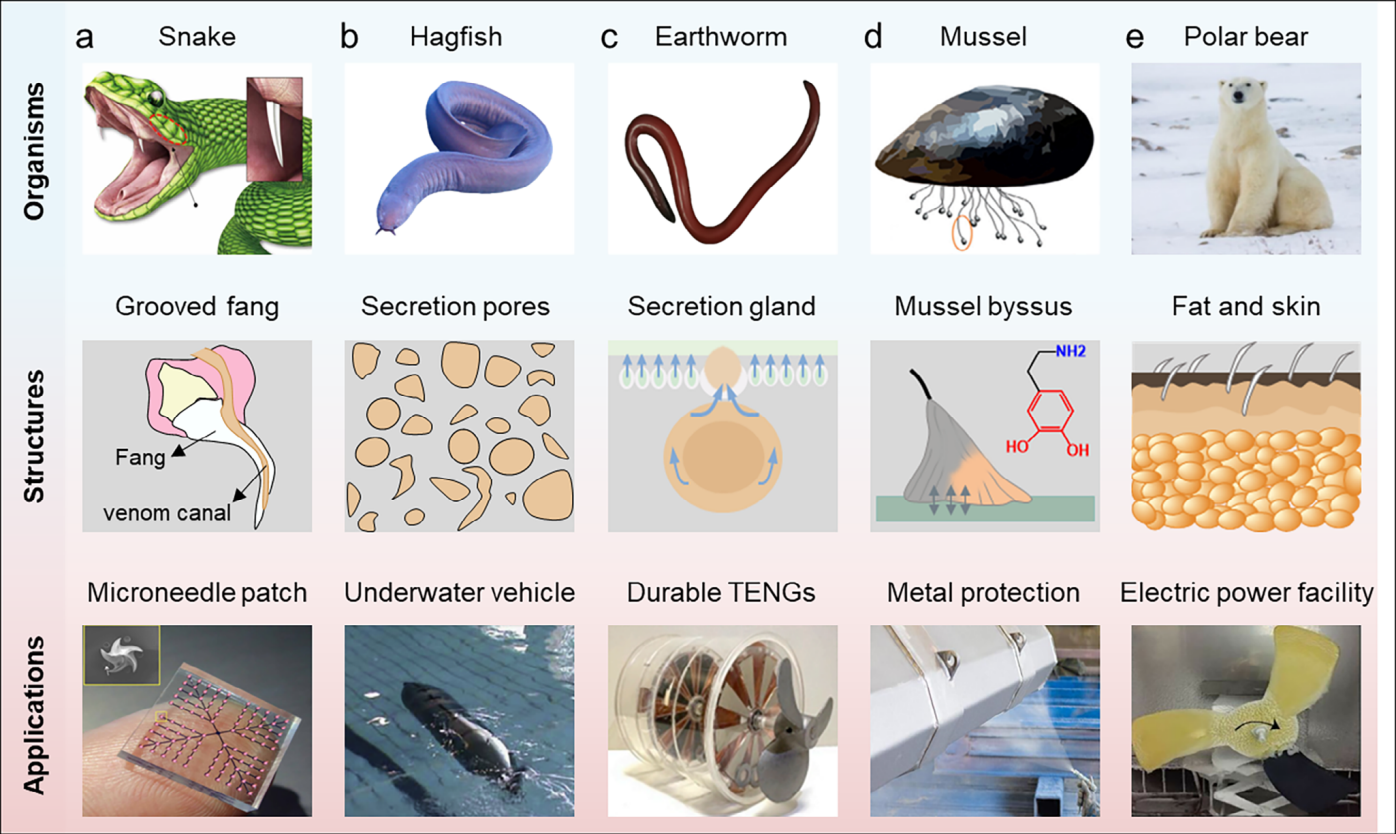
Diverse natural organisms, structures, and applications of bioinspired MICs. a) Rear‐fanged snake. Reproduced with permission.^[27a,30]^ Copyright 2019, American Association for the Advancement of Science. Copyright 2011, American Physical Society. b) Hagfish. Reproduced with permission.^[^ ^31^ ^]^ Copyright 2019, Royal Society of Chemistry. Copyright 2024, Nature. Copyright 2014, Elsevier Ltd. c) Earthworm. Reproduced with permission.^[^ ^32^ ^]^ Copyright 2024, Royal Society of Chemistry. Copyright 2020, Wiley‐VCH. Copyright 2025, Nature. d) Mussel. Reproduced with permission.^[^ ^33^ ^]^ Copyright 2024, Wiley‐VCH. e) Polar bear. Reproduced with permission.^[27b,34]^ Copyright 2022, Wiley‐VCH. Copyright 2025, Elsevier Ltd.

Living creatures in nature have a variety of ingenious structures and functions that serve as valuable sources for designing novel MICs.^[^ ^35^ ^]^ This section highlights some structural features on biological surfaces that are utilized to prepare novel MICs. The underlying principles and potential applications of these structural features are also briefly explained.^[^ ^36^ ^]^ For example, rear‐fanged snakes can rapidly inject venom or saliva through an open groove on the fang surface when they penetrate into tissue (**Figure** **3**a). Inspired by this mechanism, a microneedle patch with multiple open groove architectures capable of ultrafast transdermal delivery of liquid phase therapeutics was designed and prepared.^[^ ^27^, ^30^ ^]^ Similarly, many natural creatures can secrete mucus when exposed to diverse stimuli such as light and mechanical force. The hagfish, for example, has many micro‐scale mucus secretion pores on its skin surface containing mucus storage cavities. When exposed to light or mechanical stimuli from predators, these pores could secrete mucus to help them escape quickly. This feature provides valuable principles for applying MICs in drag reduction of underwater instruments and anti‐fouling of marine vessels. (Figure 3b).^[^ ^31^, ^37^ ^]^ Earthworms with highly developed epidermal glands can continuously secrete mucus from dorsal pores and columnar epithelial cells in response to external mechanical stimuli. Therefore, advanced materials with self‐replenishing lubrication ability have been developed to achieve the goals of anti‐friction, anti‐wear, and anti‐fouling (Figure 3c).^[^ ^20^, ^32^, ^38^ ^]^ Poison dart frogs have two types of specialized glands in their skin, which can continuously secrete mucus to moisturize the skin and secrete the toxin to deter predators.^[^ ^39^ ^]^ Seaweed, such as Saccharina japonica, is protected by a thin layer of mucus that contains and bonds large amounts of water.^[^ ^40^ ^]^ Chinese yam can secret sticky and slippery mucin, which plays an important role in water retention.^[^ ^41^ ^]^ In addition to mucus release, the strong adhesion abilities of catechol and amine in mussel adhesion proteins also provide many inspirations for designing MICs (Figure 3d). Microcapsules functionalized by polydopamine (PDA) can give the controlled release property in the self‐healing anti‐corrosive coating due to the pH sensitivity of PDA.^[^ ^33^, ^42^ ^]^ Furthermore, the fat and skin of polar bears undergo coordinated thermal management that allows them to survive in extremely cold environments, as shown in Figure 3e. The fat layer can release the stored energy in the form of heat to keep the body warm. Black skin can absorb sunlight and efficiently convert solar energy into heat energy. Inspired by polar bears, PCMCs and superhydrophobic photothermal coatings are combined to prepare sustainable anti‐icing coatings.^[^ ^27b^ ^]^ Taken together, the inherent structural features of these natural creatures can provide numerous advanced design concepts for preparing MICs. Consequently, various stimulus‐responsive microcapsules and coating materials with novel structures and functions are constantly being developed, which are used to prepare MICs with self‐regulation, self‐adaptation, self‐healing, and other fascinating capabilities. These MICs exhibit diverse performance characteristics that are crucial for their practical applications. Accordingly, we discussed about some critical properties of MICs in **Table** **2**.

**Table 2:** Comparison of performance indicators of MICs.

Stimuli	Samples	Response time	Release kinetics	Durability	Environmental stability	Refs.
Light	MICs	Temperature rises to 70 °C after 250 s; After 600 min, 90% BTA was leached	Controlled release	Not corroded within 15 d	Good self‐healing ability; Good corrosion resistance	[43]
	Pure epoxy coating	/	/	Corroded within 1 d	Poor corrosion resistance	
	MICs	Complete degradation within 4 h	Release rate increases with the increase of TiO_2_ content	Still has the self‐healing ability after 9 d	Long‐term anti‐aging	[44]
	Silicon resin	/	/	No self‐healing ability	Poor anti‐aging ability	
Mechanical	MICs	Release immediately after damage	Sustained release	Not corroded within 7 d	High temperature resistance; Good self‐lubricating property	[45]
	Pure epoxy sample	/	/	Corroded within 1 d	Poor self‐lubricating property	
	MICs	Release immediately after damage	/	Not corroded within 115 d	Good corrosion resistance	[46]
	Pure polyurea coating	/	/	Severe corrosion after 72 h	Poor corrosion resistance	
Thermal	MICs	Rapid release	/	Good bending resistance; Good washing fastness; Good dry friction fastness	Good thermal stability performance; Good adhesion resistance	[47]
	Untreated fabric	/	/	Short temperature control time	Poor thermal stability performance	
	MICs	/	/	Not corroded within 1 d; Good adhesion strength after 48 h	Good adhesion resistance	[48]
	Pure epoxy coating	/	/	Corroded within 1 d	Poor adhesion resistance	
Magnetic	MICs	/	/	Not corroded >30 d; The adhesion rate is 1%‐2%	Good anti‐fouling; Outstanding corrosion resistance	[49]
	Pure polyurethane coating	/	/	Not corroded within 96 h; The adhesion rate is 31.55%	Poor corrosion resistance; Poor antibacterial	
pH	MICs	Rapid release at first	Sustained release	Good salt spray resistance; Not corroded within 30 d	Good scale inhibition; Good corrosion resistance	[19b]
	Pure polyurea coating	/	/	Quickly corroded	Poor scale inhibition; Poor corrosion resistance	
	MICs	Rapid release at first	Release curve was pH dependent	Not corroded within 20 d	Excellent corrosion resistance	[50]
	Pure epoxy coating	/	/	Corroded within 4 d	Poor corrosion resistance	
Ion	MICs	Rapid release	Release rate increases with the increase of ion concentration	Good salt spray resistance; High temperature resistance	Excellent corrosion resistance; Stable at 25–550 °C	[51]
	Iron nail	/	/	Corroded within 7 d	Poor corrosion resistance	
	MICs	Released very quickly in 3.5 wt% NaCl solution	Sustained release	Not corroded after 72 h	Good corrosion resistance	[52]
	Carbon steel	/	/	Corroded after 6 h	Poor corrosion resistance	
Multi‐response	MICs	Rapid release	Release rate increases with the increase of acidity; The release rate increases under NIR irradiation	Not corroded within 30 d	Corrosion resistance; Weather resistance	[53]
	Pure copper	/	/	Corroded within 24 h	Poor corrosion resistance	

## 3 Material Design Criteria of MICs

### 3.1 Shell Materials

**Figure 4:**
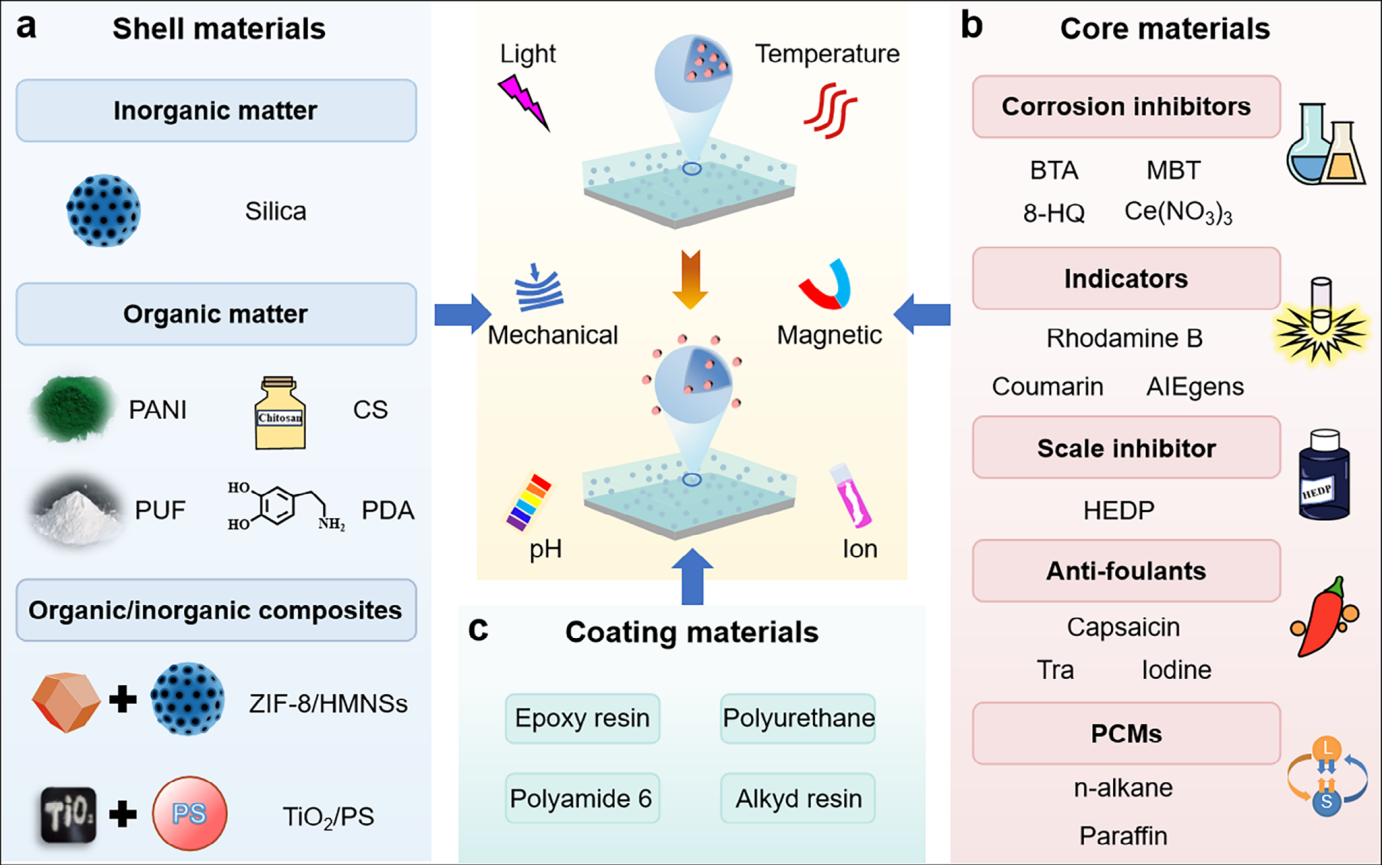
Selections of shell, core, and coating materials in MICs. a) Shell materials of microcapsules, including inorganic matter, organic matter, and organic/inorganic composites. b) Core materials of microcapsule such as corrosion inhibitors, indicators, scale inhibitor, anti‐foulants, and phase change materials (PCMs). c) Coating materials such as epoxy resin, polyurethane (PU), polyamide 6 (PA6), and alkyd resin.

The shell of microcapsules plays a critical role in isolating the core material from the external environment.^[^ ^54^ ^]^ Therefore, the stability of shell materials should be a top priority. To achieve the controlled release of the loadings, an intelligent permeability of shell materials is another important factor for designing MICs. Furthermore, the compatibility of shell materials with the coating materials also decides the performance of MICs. In summary, the stability, permeability, and compatibility with coatings should be considered when selecting shell materials for preparing MICs. Diverse inorganic matter, organic matter, and their hybrid composites have been selected as shell materials to prepare microcapsules (**Figure** **4**a).

#### 3.1.1 Inorganic Matter

Silica (SiO_2_) has been widely used as an inorganic shell material for preparing microcapsules due to its numerous advantages such as easy surface modification, large coverage area, and good stability.^[^ ^6b^ ^]^ Different loadings such as paraffin, fluorescent agents, and ionic liquids can be encapsulated within SiO_2_ shells for obtaining MICs with advanced properties. For example, paraffin@SiO_2_‐based PCMCs exhibit an 80.7% enhancement in thermal conductivity and better compatibility with polydimethylsiloxane (PDMS) substrate compared with their non‐paraffin counterparts.^[^ ^55^ ^]^ Fluorescent agents/cationic photo‐initiator@SiO_2_ microcapsules was prepared by a combined interfacial/in situ polymerization, yielding systems with self‐reporting and self‐healing functions.^[^ ^56^ ^]^ Ionic liquids@SiO_2_ was prepared to obtain high‐temperature resistant microcapsules.^[^ ^57^ ^]^ To sum up, the inorganic microcapsules have stable physicochemical and mechanical properties. However, the interfacial compatibility between inorganic shells and polymer materials is poor, leading to undesirable aggregation in coatings.^[^ ^58^ ^]^ Methods such as grafting polymer chains onto the surface of inorganic matter and blending the interfacial compatibilizer with the systems have been used to improve the compatibility between inorganic matter and organic matter.^[^ ^59^ ^]^ Consequently, optimizing interfacial compatibility by using the above methods can lead to MICs with superior performance when using inorganic microcapsules.

#### 3.1.2 Organic Matter

During the fabrication of microcapsules, natural polymers, synthetic polymers, and naturally modified synthetic polymers have been widely employed as shell materials.^[^ ^60^ ^]^ Among natural polymers prepared for shell materials, the commonly used materials are chitosan and alginate due to their advantages of biocompatibility, biodegradability, and non‐toxicity.^[^ ^61^ ^]^ Additionally, the combination of sodium alginate and chitosan is one of the most studied microcapsule systems.^[^ ^62^ ^]^ For example, linseed oil/1H‐Benzotriazole (BTA)@sodium alginate/chitosan microcapsules were prepared as responsive microcapsules with a low impact on the environment.^[^ ^63^ ^]^ However, natural polymeric shells have certain limitations, such as poor stability, a tendency to swell, and mechanical fragility, which restrict their long‐term use. The selection of synthetic polymers as microencapsulated shells can overcome these drawbacks to a certain extent.^[^ ^61b^ ^]^ Chen's team prepared nanocapsules with polycaprolactone‐polyethylene glycol‐folic acid (PCL‐PEG‐FA) as the shell by using microfluidic technology. The prepared nanocapsules not only have biocompatibility, but also have adjustable size and shell thickness, high packaging efficiency, and loading capacity.^[^ ^64^ ^]^ Many polymers, such as polysulfone (PSF), polyurea‐formaldehyde (PUF), and polyaniline (PANI) have been used as microcapsule shells. PSF and PUF have good mechanical properties and permeability, which play key roles in protecting the core materials and extending the service life of microcapsules.^[^ ^65^ ^]^ For example, the microcapsules with PUF as the shell can act in the marine environment for up to four months.^[^ ^65a^ ^]^ The friction coefficient of coatings reduced by 87.9% with 10 wt% PAO40@PSF microcapsules added.^[^ ^66^ ^]^ PANI, a dense film, can not only prevent the leakage of the core material but also endow MICs with high thermal conductivity, photothermal conversion, and anti‐corrosion functions.^[^ ^19^, ^67^ ^]^ PDA is often used to modify the polymer shells to improve the mechanical properties, thermal stability, and oxidation resistance of microcapsules due to its high adhesion and biocompatibility.^[^ ^68^ ^]^ In conclusion, organic microcapsules offer multi‐functional and customizable properties, however, these properties are susceptible to environmental factors, resulting in reduced stability and durability of microcapsules. For use in complex environments, combining inorganic components with organic substances into a single shell is an effective way for preparing advanced microcapsules, as discussed below.

#### 3.1.3 Inorganic/Organic Composites

A typical organic/inorganic shell of microcapsules consists of a hollow organic shell covered with inorganic nanoparticles. It is common to coat the shell (e.g., polystyrene (PS), PUF, and PSF) with SiO_2_ or TiO_2_ nanoparticles. For example, pH‐responsive jasmine@SiO_2_/PS microcapsules were prepared via Pickering emulsion polymerization, which can be used for the preparation of functional textiles.^[^ ^69^ ^]^ Silica/polyurea hybrid shells have been used to prepare microcapsules by interfacial polymerization and in situ polymerization for improving their storage stability and self‐healing ability.^[^ ^70^ ^]^ In addition, organic hollow shells loaded with TiO_2_ nanoparticles have also been used as shell materials to prepare advanced microcapsules. For instance, PUF/TiO_2_ hybrid shells were used to prepare microcapsules with photo‐absorbing properties.^[^ ^71^ ^]^ Healing agent@polymer/TiO_2_ microcapsules were fabricated via UV‐initiated polymerization of Pickering emulsions for obtaining light and mechanical responsive microcapsules.^[^ ^44^ ^]^ Another typical organic/inorganic shell of microcapsules is an inorganic porous shell, such as hollow mesoporous silica nanoparticles (HMSNs) coated with metal‐organic frameworks (MOFs). For example, ZIF‐8, a well‐known pH‐responsive MOF, exhibits excellent chemical and thermal stability, high water stability, and pH reactivity.^[^ ^72^ ^]^ The superior compatibility and uniform dispersion behavior of BTA@HMSN/ZIF‐8 in the epoxy resin coatings are attributed to the existence of ZIF‐8.^[^ ^72^ ^]^ In addition, enhanced microbiological corrosion protection properties of coatings were obtained by coating ZIF‐8/MNZ@HMSNs‐water borne alkyd resin onto the substrates, demonstrating corrosion potential of −0.54 V/SCE for the coated substrates compared with −0.65 V/SCE for blank substrates.^[^ ^73^ ^]^ Furthermore, Chen and his colleagues developed BTA@HMSN/ZIF‐8 nanocapsules with bactericidal and corrosion inhibition functions. Zinc ions in ZIF‐8 can react with S^2−^ ion to form stable ZnS, which greatly improves the stability of the mentioned nanocapsules.^[^ ^51^ ^]^ In summary, by combining the advantages of organic and inorganic shells, microcapsules display excellent physicochemical properties and intelligent responsive functions, resulting in higher compatibility with coating matrices. MICs with advanced properties can be obtained using this formulation, offering a wide scope for the development of the application of microcapsules.

### 3.2 Core Materials

By loading corrosion inhibitors, fluorescent indicators, scale inhibitors, anti‐foulants, and PCMs into microcapsules as core materials (Figure 4b), MICs can perform diverse functions and be used in various fields. Consequently, the selection of core materials loaded in microcapsules is of great importance for optimizing the performance of MICs. This section will present and discuss in detail based on the types of core materials.

#### 3.2.1 Corrosion Inhibitors

Corrosion inhibitors used in MICs can be categorized into inorganic and organic corrosion inhibitors. Inorganic corrosion inhibitors mainly include cerium‐^[^ ^74^ ^]^ and chromium‐based^[^ ^75^ ^]^ inhibitors. Taking cerium phosphate^[^ ^74a^ ^]^ and cerium nitrate^[^ ^76b–d^ ^]^ for example, cerium ions can migrate to the corrosion site and react to form a protective layer of oxides or hydroxides to prevent corrosion. It should be noted that chromium‐based corrosion inhibitors have the risk of releasing hexavalent chromium, and this risk could be mitigated by means of valence state stabilization treatment to reduce environmental toxicity. In contrast, organic corrosion inhibitors mainly include sulfur, oxygen, or nitrogen heterocyclic structures of organic compounds, such as BTA,^[^ ^23^, ^42^, ^72^, ^76^ ^]^ 2‐mercaptobenzothiazole (MBT),^[^ ^77^ ^]^ and 8‐hydroxyquinoline (8‐HQ).^[^ ^21^, ^24^, ^67^, ^78^ ^]^ These organic inhibitors can be adsorbed on the corroded metal surface through electrostatic interactions or coordination complexes, ultimately achieving excellent anti‐corrosion function. However, such heterocyclic compounds need to be evaluated for their biodegradability and ecological accumulation effects. Recent studies showed that the half‐life of 8‐HQ in simulated aquatic environments can exceed 120 d.^[^ ^79^ ^]^ More importantly, protection against metal corrosion is usually accompanied by the detection and reporting of early damage. This requires microcapsules loaded with some fluorescent indicators, such as AIEgens,^[^ ^80^ ^]^ coumarin,^[^ ^81^ ^]^ 1,10‐phenanthroline,^[^ ^82^ ^]^ and Rhodamine B.^[^ ^83^ ^]^ The introduction of fluorescent indicators endows MICs with self‐reporting functions at an early stage of corrosion, enabling better monitoring of the corrosion process and timely initiation of appropriate material maintenance to extend the life of the coatings and metals. It is necessary to consider the potential secondary pollution caused by the photo‐degradation products of fluorescent dyes. Therefore, rhodamine B derivatives that can be photo‐catalytically decomposed are well candidates. Taken together, the indicators and corrosion inhibitors can act synergistically to delay the corrosion of materials. By applying MICs onto the surface of diverse materials, especially for metal, excellent and intelligent anti‐corrosion materials can be obtained and used in many fields such as industrial equipment and vehicles. More importantly, depending on different application needs, the customized formulation of MICs with anti‐corrosion properties can be designed and prepared according to their advantages and disadvantages shown in **Table** 3, which is important for the development of MICs.

#### 3.2.2 Scale Inhibitors

Scale inhibitors are a kind of agent that can disperse insoluble inorganic salts in water and prevent or affect the precipitation and deposition of insoluble inorganic salts on the metal surface.^[^ ^84^ ^]^ These compounds exhibit excellent resistance to weak acid, strong alkaline, good scale resistance, while also demonstrating good thermal conductivity. 1‐Hydroxyethylidene‐1,1‐diphosphonic acid (HEDP) is a kind of organic phosphonic acid scale inhibitor, which can form stable complexes with iron, copper, zinc, and other metal ions, and dissolve oxides on the metal surface.^[^ ^85^ ^]^ Notably, HEDP can maintain its anti‐deposition effect even at temperature up to 250 °C. Wang's group added HEDP to the porous microcapsules, and when the scale was deposited on the metal surface, a stable intermediate of HEDP:Ca^2+^ was formed. Due to the release of HEDP, the scale inhibition efficiency was up to 78.57%.^[^ ^19b^ ^]^ In summary, the use of scale inhibitors can effectively prevent or reduce the deposition and scaling of insoluble inorganic salts. Although HEDP is an efficient scale inhibitor with significant advantages such as high chemical stability, excellent anti‐scaling performance, and good adaptability to harsh environments, its environmental risks cannot be ignored. HEDP shows obvious toxicity to aquatic organisms, has potential bioaccumulation, and inhibits environmental microorganisms. Furthermore, HEDP shows high persistence in the environment due to the thermodynamically stable carbon‐phosphorus bonds and microbial inhibition. The degradation bottleneck of HEDP may be broken through the physicochemical‐biological synergistic process.^[^ ^86^ ^]^ More importantly, the development of more efficient and eco‐friendly scale inhibitors is urgent for advanced MICs with anti‐scaling properties.

#### 3.2.3 Anti‐Foulants

In order to achieve the effect of anti‐fouling, incorporating anti‐foulants into microcapsules is essential. Capsaicin, extracted from natural peppers without disrupting biological chains, can be used as a marine anti‐fouling agent to kill plant spores and animal larvae attached to the outer surface of ships.^[^ ^65a^ ^]^ Additionally, tralopyril (4‐mo‐2‐(4‐chlorophenyl)‐5‐(trifluoromethyl)‐1H‐pyrrole‐3‐carbonitrile (Tra)), an eco‐friendly anti‐foulant, can also be used in a new type of slippery liquid infused porous surface (SLIPS) for marine anti‐fouling applications.^[^ ^87^ ^]^ Moreover, iodine is highly effective as a broad‐spectrum anti‐microbial agent with no reported resistance, making it suitable for preventing cross‐contamination.^[^ ^5^ ^]^ In summary, anti‐foulants have broad application prospects in marine and medical devices. However, when used in medical devices, MICs must have good biocompatibility. Therefore, materials and anti‐foulants approved by the Food and Drug Administration (FDA) should be employed to prepare MICs for medical devices.

#### 3.2.4 PCMs

PCMs are known for their ability to store or release a large amount of latent heat during phase transition,^[^ ^55^ ^]^ showing important applications in fields such as anti‐icing/de‐icing,^[^ ^27^, ^88^ ^]^ smart textiles,^[^ ^89^ ^]^ and green buildings.^[^ ^90^ ^]^ Encapsulating PCMs into microcapsules to isolate them from the external environment can reduce leakage to some extent.^[^ ^28^, ^55^ ^]^ Currently, the core PCMs for microcapsules in MICs are usually long‐chain alkanes, such as paraffin,^[^ ^89^, ^91^ ^]^ *n*‐tetradecane,^[^ ^27^, ^88^ ^]^ *n*‐octadecane,^[^ ^92^ ^]^ and *n*‐eicosane.^[^ ^18^, ^88^ ^]^ For example, when paraffin@SiO_2_ microcapsules were added to PDMS, the latent heat value of the material increased to 60.61 J g^−1^, and its thermal stability improved by 80.71%.^[^ ^55^ ^]^ These PCMs have temperature stability, environmental protection, and controllability, so they have great application value and potential in thermal management, anti‐icing, and de‐icing. Among these, paraffin stands out due to its low cost, high latent heat capacity, and high transformation temperature, leading to its wide use in PCM formulations.

#### 3.2.5 Lubricants

Various lubricants as the core of the microcapsules can help MICs achieve excellent self‐lubricating and self‐replenishing properties. Firstly, ionic liquids have proved to be excellent lubricating oils with excellent thermal and oxidative stability. PA6 composites with 3 wt% IL@SiO_2_ nanocapsules showed a 38.9% decrease in friction coefficient and a 60.7% decrease in wear rate compared with pure PA6. Therefore, it can be used as an ideal material for preparing high‐temperature resistant coatings.^[^ ^57^ ^]^ Secondly, linseed oil has similar viscosity and thermal stability to lubricating oil, which is also a popular lubricant widely used in the design and development of self‐healing and self‐lubricating coatings.^[^ ^93^ ^]^ Then PAO40, as a synthetic oil, is receiving more and more attention due to its low cost and good lubrication performance.^[^ ^68a^ ^]^ Finally, tung oil, as an excellent self‐healing agent, has good film formation when contacted with oxygen and has excellent thermal stability and a viscosity similar to that of lubricating oil, so it can also be added to microcapsules as a self‐healing agent and self‐lubricant.^[^ ^94^ ^]^ When 10 wt% tung oil‐loaded microcapsules were added to the epoxy resin, the friction coefficient and wear rate were reduced by 17.3% and 78.6% compared with the pure epoxy resin. Taken together, the use of these lubricants in MICs can endow the surface with good lubricating properties. As listed in Table 3, each lubricant has its own advantages and disadvantages. To maximize the performance of MICs, choosing the appropriate lubricant is important and essential for different applications.

In conclusion, the selection of core materials in microcapsules plays a key role in MICs. The core materials can not only determine the functionality and practicality of the coating but also affect the performance and application scenarios of the coating. Therefore, great attention should be paid to the selection of microcapsule loadings in the design and preparation of MICs to ensure optimal performance tailored to intended uses.

### 3.3 Coating Materials

When selecting coating materials, many factors, including environmental impact, service life, economic efficiency, environmental protection, and compatibility, must be considered. Organic coatings are among the most common coating types, including epoxy resin, PU, PA6, and alkyd resin (Figure 4c). Epoxy resin is widely used due to its excellent mechanical properties, easy processing, and excellent chemical resistance.^[^ ^95^ ^]^ It serves as a substrate for various coatings, such as anti‐corrosion coatings,^[^ ^24^, ^63^ ^]^ self‐reporting coatings,^[^ ^80^, ^82^ ^]^ and self‐lubricating coatings.^[^ ^93^, ^94^ ^]^ Notably, epoxy resin can be used as a shape memory polymer (SMP), which deforms under external stimulation and returns to its initial shape after re‐stimulation.^[^ ^43^ ^]^ However, epoxy resin has disadvantages such as poor weather resistance and low impact resistance. These drawbacks are often mitigated by adding some particles as fillers. For example, Wang et al. improved the performance of epoxy resin by adding carnauba wax microparticles as fillers to prepare self‐healing shape memory composite coatings.^[^ ^96^ ^]^ When the temperature was raised, the melted wax could flow and seal the remaining defects when recrystallized to ensure complete barrier protection, giving these coatings excellent healing properties. According to the characteristics of wear resistance, aging resistance, and high elasticity, PU is an established environmentally friendly coating material for MICs.^[^ ^97^ ^]^ Similar to epoxy resin, PU can be designed as an SMP that can be triggered by NIR light for shape memory recovery. PA6, as a polymer with easy forming, wear resistance, and high mechanical strength, is also often used as a substrate in intelligent self‐lubricating coatings.^[^ ^57^, ^68^ ^]^ In addition, alkyd resin^[^ ^42^ ^]^ and PDMS^[^ ^55^ ^]^ are also used as coating materials in MICs because of their inherent properties. As detailed in Table 3, these polymer materials each have distinct advantages and disadvantages, making them suitable for different applications. Consequently, selecting the appropriate substrate is a critical consideration during the design and preparation of MICs.

In summary, we have introduced the commonly used shell, core, and coating materials for previously fabricated MICs. A wide range of materials is available for designing MICs. To provide a more suitable material selection for the design and development of MICs, we list various materials with excellent performance, their advantages and disadvantages in Table **3**, and also their scopes of application, which can provide guides for researchers to design and develop novel MICs.

**Table 3:** Summary of commonly used shell, core, and coating materials in preparing MICs.

Types	Classification	Materials	Advantages	Disadvantages	Applications	Refs.
Shell	Inorganic shell	Silica	Easy surface modification; Large specific surface area; Adjustable morphology and structure	Single function; Biological toxicity; Poor degradation performance	Drug carrier; Catalytic carrier; Semiconductor material	[55, 56, 57]
	Organic shell	CS	Biocompatibility; Biodegradability	Weak anti‐oxidant; Poor water solubility; Poor mechanical properties	Dressings; Medical film; Water treatment	[63]
		Alginate	Good solubility; Biocompatibility; Biodegradability	Short service time; Poor elasticity and mechanical properties	Hydrogels; Coating material; Wound dressings	[63]
		PSF	High creep resistance; High mechanical properties; High temperature resistance	Easy to crack; Poor liquidity; Difficult to process	Biological medicine; Electronic appliance; Water treatment membrane	[65b]
		PUF	Low cost; Good thermal stability and compatibility	Toxicity; Difficult to process;	Coating; Adhesive;	[65a]
		PANI	High extinction coefficient; Excellent thermal conductivity; Strong near infrared absorption	Poor dispersion; Poor solubility; Poor membrane tightness	Conductive material; Water treatment material; Metal corrosion protection	[19, 67]
		PDA	High adhesion; Biocompatibility; Photothermal conversion	Long deposition time; Poor stability and uniformity	Nanomedicine; Bio‐detection and imaging; Material surface modification	[68a,b]
		PS	Good solubility; Low viscosity	Poor heat resistance; Poor mechanical strength	Various encapsulation materials	[98]
	Organic/inorganic shell	PU/SiO_2_	Easy to synthesize; High thermal stability; Excellent mechanical properties	Easy to assemble; Poor biocompatibility	Good encapsulation material	[70]
		PSF/SiO_2_				[99]
		PS/SiO_2_				[69]
Shell	Organic/inorganic shell	TiO_2_/PUF	High photocatalytic activity; Good biocompatibility/chemical stability	High cost; Easy cluster; Environmental pollution	Self‐cleaning; UV protection agent; Anti‐bacterial/anti‐fouling paint	[71]
		TiO_2_/PS				[44]
		Silica/ZIF‐8	Easy synthesis; Large specific surface area; Good pH sensitivity, thermal stability, and biocompatibility	Poor recyclability; Difficult to separate	Controlled release; Good encapsulation material	[51, 72]
		Silica/PDA				[42]
		Silica/CS				[82]
Core	Corrosion inhibitor	Cerium salt	Cheap; Low toxicity; Good corrosion inhibition effect	Selective on substrates	Anti‐corrosion for metals	[74, 75]
		BTA	Can be used with a variety of corrosion; Corrosion resistant to a variety of metals	Toxicity; Poor corrosion inhibition at low pH value	Anti‐corrosion for metals	[23, 76]
		8‐HQ	High temperature resistance; Strong acid and alkali resistance	Toxicity; Corrosion resistant only to aluminum alloy	An efficient inhibitor and sensing probes for aluminum ions	[21, 78]
		MBT	Non‐toxicity; Non‐polluting; Good selection for adsorption	Poor water solubility; Poor chlorine resistance	Anti‐corrosion for copper; Industrial water treatment	[77]
	Indicator	AIEgens	High brightness; Strong light stability; Good biocompatibility	Synthetic complexity; High environmental toxicity	Bioimaging; Fingerprint detection; Photodynamic therapy	[80]
		Coumarin	Good light stability; Easy modification; High fluorescence quantum yield	Limited brightness; Overlap with the autofluorescence signal	Early damage report of metals	[81]
		1,10‐phenanthroline	Cheap; High sensitivity; Wide range of applications	Toxicity; Pollute the environment	Metal detection; REDOX indicator	[82]
Core	Indicator	Rhodamine B	Strong brightness; Excitation and emission wavelengths are in the visible spectrum	Affected by pH; Poor light stability	Early damage report of metals	[83]
	Scale inhibitor	HEDP	Good anti‐scaling; High chemical stability; Suitable for harsh environment	Toxicity; Pollute the environment	Anti‐scaling for steel equipment	[19b]
	Anti‐foulant	Capsaicin	Non‐toxicity; Non‐polluting	Fast leakage speed; Short action period	Anti‐phlogistic drug; Marine anti‐fouling coating	[65a]
		Tra	Eco‐friendly	Limited scope	Marine anti‐fouling coating	[87]
		Iodine	Efficient; Broad spectrum	Low solubility; Easy sublimation	Anti‐fouling coatings for medical devices	[5]
		MNZ	Cheap; Good anti‐bacterial effect	/	Anti‐bacterial drug	[51, 73]
	PCM	Paraffin	Cheap; High latent heat; High transformation temperature	Inflammable; Low thermal conductivity	Heat storage capacity for use in thermal management	[91]
		*n*‐Tetradecane	Non‐toxic; High solution heat; Good thermal conductivity	Untimely phase transition	Anti‐icing/de‐icing under low temperature; Temperature control of infrared stealth materials	[88a]
		*n*‐Octadecane				[92]
		*n*‐Eicosane				[88b]
	Lubricant	Ionic liquid	High thermal stability; Excellent oxidation resistance; Low steam pressure and volatility	Corrosion; High cost; Thermal oxidation	Self‐lubricating; High temperature processing	[57]
		Linseed oil	Cheap; Air‐drying; Environmentally friendly	Easy to oxidize; Easy hydrolysis	Self‐lubricating	[93d]
		PAO40	Low cost; Good lubrication properties; Excellent high/low temperature performance	Poor solubility; Poor friction resistance	lubricating oil; Refrigerating oil; High temperature aviation	[68a]
Core	Lubricant	Tung oil	Wear‐resistant; Excellent thermal stability	Poor weather resistance; Aging easily	Waterproof, anti‐corrosion, anti‐rust coating	[94]
Coating	Polymer coating	Epoxy resin	Strong adhesion; Good mechanical properties; High temperature resistance	Low impact strength; Poor weather resistance	Coating and adhesive; Engineering material; Shape memory material	[96]
		Polyurethane	Good elasticity; Environmental protection; Wear and aging resistant	High cost; Poor flame retardancy; Unstable in strong acid/alkali	Waterproof coating; Shape memory material; Thermal insulation material	[97]
		PA6	Good toughness; High mechanical strength; Wear/corrosion resistance	Easily absorb water; Poor light resistance	Thin film Automobile industry; Electrical/electronic industry	[57, 68]
		PDMS	Low cost; Excellent flexibility; Chemical inertness	Easy to deformation; Poor thermal conductivity; Highly hydrophobic surface	Wearable sensor; Flexible substrate; Microfluidic chip materials	[55]
		Alkyd resin	Excellent flexibility; Water/heat resistance; Physical/mechanical properties	Poor resistant to alkali	Paint; Coating	[42]

Abbreviations: CS: chitosan; PANI: polyaniline; PDA: polydopamine; PUF: polyurea‐formaldehyde; PSF: polysulfone; PS: polystyrene; PU: polyurea; BTA: 1H‐Benzotriazole; 8‐HQ: 8‐Hydroxyquinoline; MBT: 2‐Mercaptobenzothiazole; HEDP: 1‐Hydroxyethylidene‐1,1‐diphosphonic acid; Tra: 4‐mo‐2‐(4‐chlorophenyl)‐5‐(trifluoromethyl)‐1H‐pyrrole‐3‐carbonitrile; MNZ: metronidazole; PA6: polyamide 6; PDMS: polydimethylsiloxane.

## 4 External Stimulus Responses for Triggering MICs

The microcapsules and coating matrixes in MICs can sense changes in the external environment and respond accordingly in a controlled manner. MICs can be categorized into different responsive types based on stimulus sources, including light, mechanical, temperature, magnetic, pH, ion, and multiple responses. This section briefly discusses these responsive MICs, highlighting their advantages and disadvantages in detail (**Table** **4**).

**Table 4:** Summary of MICs triggered by different stimuli, including light, mechanical, thermal, magnetic, ion, pH, and multiple stimuli.

Stimuli	Conditions	Materials	Advantages	Disadvantages	Applications	Refs.
Light	UV	TiO_2_	Clean; Easy to obtain; Remote control; Precision control	Biotoxicity; Susceptible to light scattering	Drug release; Photochromic; Controlled catalysis; Functional coating	[4, 44]
		Azo				[100, 112]
		PDA				[109b]
	NIR	TiN				[43]
		*o*‐nitrobenzyl				[108a]
		SMPU				[97]
Mechanical	Stretching	PUF	Instant; Efficient; Reversible; Easily accessible	Small selection of materials	Drug release; Self‐healing coating; Damage visualization coating	[68b]
	Pressure	CS/SiO_2_				[102a]
	Osmotic pressure	PEGDA				[102b]
	Friction	PSF				[45]
		PS				[122]
Thermal	T < 34 ^o^C	PDEAM	Ease of operation; Short response time; Adjustable transition temperatures	Easily affected by environmental temperature	Hydrogel; Industrial catalysis; Drug controlled release	[125]
	T > 4∼9 ^o^C	*n*‐tetradecane				[18e]
	T > 32 ^o^C	PVCL				[103]
	T > 35 ^o^C	*n*‐eicosane				[47]
	T > 37 ^o^C	P(NAGAm‐co‐NPhAm)				[126]
	T > 82 ^o^C	SMPU				[128]
	T > 82 ^o^C	SMEP				[129]
	T > 82 ^o^C	EVA				[74d]
	T > T_g_	PMMA‐MA				[127]
Magnetic	Magnetic heating	Cobalt ferrite	Remote control; Quick response; High biosecurity; Strong penetration	Strict equipment requirements	Cell segregation; Targeted drug delivery; Nuclear magnetic imaging	[104]
	Magnetic field	Fe_3_O_4_				[49, 133]
pH	pH > 7	PANI	High sensitivity; Strong operability	Equipment corrosion; Environmental pollution	Hydrogel; Diagnose diseases; Drug controlled release	[138]
		SA				[19b]
	pH < 7	PDEAEMA				[105]
		PDA				[42]
		ZIF‐8				[24, 136]
		CS				[137b]
		Alginate				[137a]
Ion	Cl^−^	PANI	Quick response	Poor sensitivity; Environmental pollution	Anti‐corrosion; Biomedical field; Wastewater treatment	[140b]
	H^+^, OH^−^	PDDA/PSS				[77d]
	S^2−^	ZIF‐8				[51, 73]
Multiple stimuli	Light/pH (NIR/pH < 7)	PDA	Easy to obtain; Remote control; High sensitivity; Strong operability	Biotoxicity; Environmental pollution; Susceptible to light scattering	Antibacterial coating; Anti‐corrosion coating	[53, 142]
	Light/pH (UV/pH < 7)	SiO_2_/TiO_2_/PDEAEMA			Water‐based coating	[143]
	Light/pH (UV or NIR/pH > 7/pH < 7)	PDA/ZnO			Intelligent self‐healing coating	[106]
	Light/Thermal (NIR/T > *T*_g_)	TiN	Easy to obtain; Remote control; Short response time	Biotoxicity; Easily affected by environmental temperature	Anti‐corrosion coating	[43]
	Light/Thermal (NIR/T > *T*_g_)	PANI				[19c]
	Thermal/pH (T > 37 °C /pH < 7)	PNIPAM/PDEAEMA	High sensitivity; Strong operability; Ease of operation; Short response time	Environmental pollution; Easily affected by environmental temperature	Multidrug preprogrammed delivery	[144]
	Magnetic/Thermal (Magnetic field/T > 37 °C)	Iron oxide nanocrystals/PNIPAM	Remote control; Quick response; Ease of operation; Short response time	Strict equipment requirements; Easily affected by environmental temperature	Drug carriers	[130c]

Abbreviations: Azo: azobenzene; PDA: polydopamine; SMPU: shape memory polyurethane; PUF: polyurea‐formaldehyde; CS: chitosan; PSF: polysulfone; PS: polystyrene; PEGDA: poly(ethylene glycol) diacrylate; EVA: ethylene vinyl acetate; PVCL: poly‐N‐Vinylcaprolactam; SMEP: shape memory epoxy resin; PANI: polyaniline; PDDA: poly (diallyl dimethylammonium chloride); PSS: poly (styrene sulfonate); ZIF‐8: zeolitic imidazolate framework‐8; PDEAEMA: poly (2‐(dimethylamino)ethyl methacrylate); SA: stearic acid; PNIPAM:poly (N‐Isopropyl acrylamide).

### 4.1 Light Response

Light is a particularly attractive energy source with favorite features such as cleanliness, low cost, spatiotemporal, controllability, and predictability.^[^ ^107^ ^]^ Benefits from these advantages of light stimulation, it has become a perfect external stimulus to control the behavior of microcapsules and also other responsive materials.^[^ ^107^, ^108^ ^]^ In recent years, light‐triggered MICs based on light‐responsive microcapsules or coating matrixes have been prepared and made great progress in self‐healing coating,^[^ ^109^ ^]^ drug delivery,^[^ ^110^ ^]^ and other applications.

#### 4.1.1 Light Responsive Microcapsules

**Figure 5:**
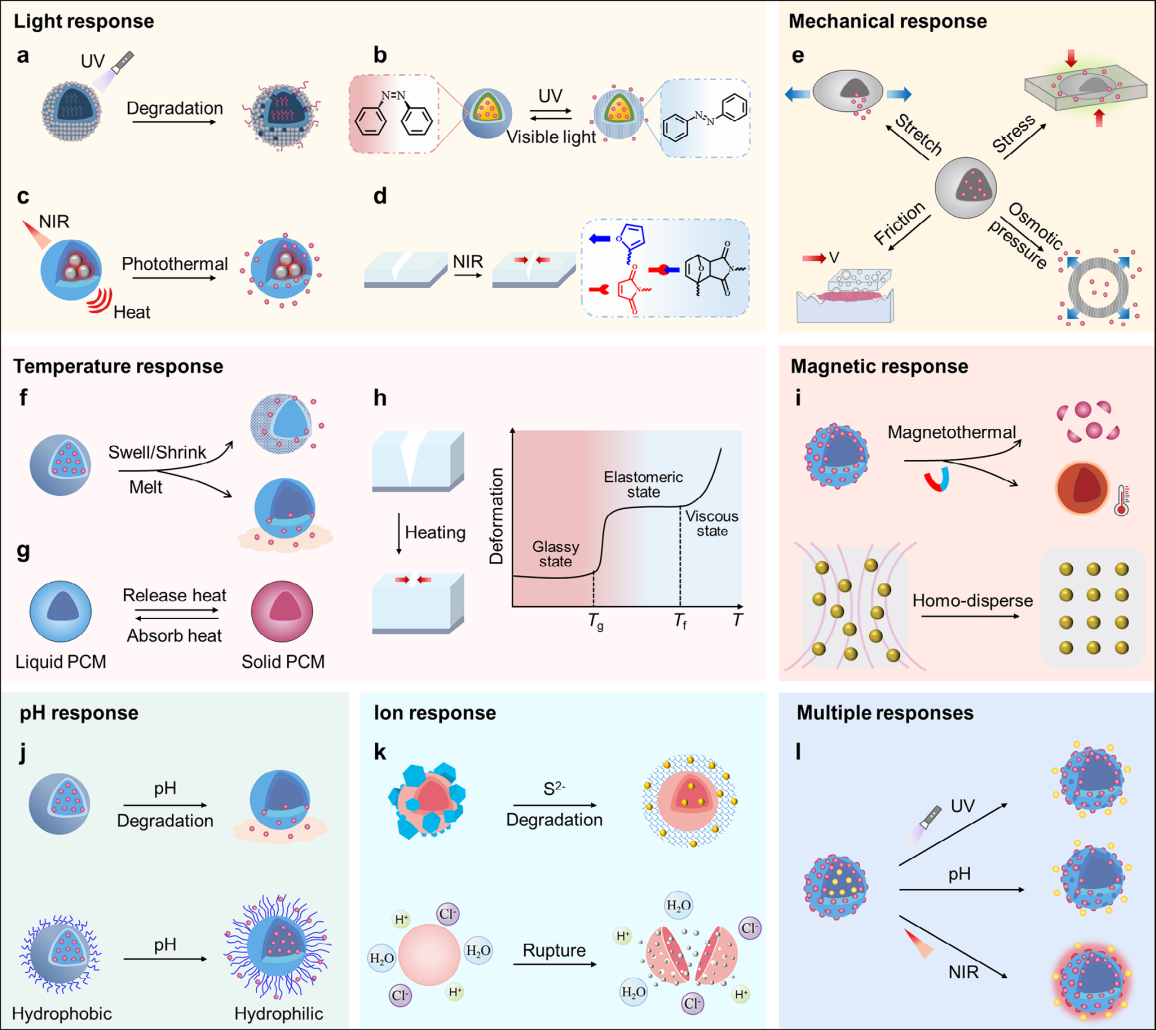
Schematic diagram of several responsive types in triggering MICs, including light, mechanical, temperature, magnetic, pH, ion, and multiple responses. a) Shell degradation induced by photocatalytic materials under UV irradiation. Reproduced with permission.^[^ ^4a^ ^]^ Copyright 2015, Royal Society of Chemistry. b) Conformational changes induced by photochromic molecules under UV or visible light irradiation. Reproduced with permission.^[^ ^100^ ^]^ Copyright 2015, Royal Society of Chemistry. c) Inhibitor release induced by photothermal materials under NIR irradiation. Reproduced with permission.^[^ ^43^ ^]^ Copyright 2021, Elsevier Ltd. d) Shape recovery mechanism of MICs under NIR stimulated Diels‐Alder (DA) reaction. Reproduced with permission.^[^ ^101^ ^]^ Copyright 2021, Elsevier Ltd. e) Mechanical stimulus response of MICs triggered by pressure, osmotic pressure, stretch, and friction, respectively. Reproduced with permission.^[^ ^45^, ^68^, ^102^ ^]^ Copyright 2021, Royal Society of Chemistry. Copyright 2013, Royal Society of Chemistry. Copyright 2020, Wiley‐VCH. Copyright 2017, Elsevier Ltd. f) Phase/volume change of the shell induced by the change of temperature. Reproduced with permission.^[^ ^74^, ^103^ ^]^ Copyright 2022, Elsevier Ltd. Copyright 2020, Elsevier Ltd. g) Phase change of the core caused by the change of temperature. h) Schematic diagram of shape recovery mechanism of shape memory coating induced by thermal‐stimulated phase transition. i) Magnetothermal effect and uniform distribution of microcapsules in response to magnetic stimulus. Reproduced with permission.^[^ ^104^ ^]^ Copyright 2020, Elsevier Ltd. j) pH‐triggered degradation or protonation of the shell. Reproduced with permission.^[^ ^105^ ^]^ Copyright 2021, Elsevier Ltd. k) Ion‐triggered degradation or rupture of the shell. Reproduced with permission.^[^ ^20^, ^73^ ^]^ Copyright 2021, Elsevier Ltd. Copyright 2021, Springer. l) The degradation and dissolution of the shell triggered by multiple responses. Reproduced with permission.^[^ ^106^ ^]^ Copyright 2021, American Chemical Society.

Light‐responsive microcapsules mainly rely on the introduction of photosensitive materials or groups into the microcapsule materials. Photosensitive materials or groups can absorb light energy to trigger photochemical reactions, resulting in the responsive MICs. Photocatalytic materials (e.g., TiO_2_ and WO_3_) and photothermal materials (e.g., titanium nitride (TiN), carbon‐based materials, and noble metals) have been used to prepare light‐responsive MICs. (**Figure** **5**a,b).^[^ ^4^, ^6^, ^43^, ^44^, ^111^ ^]^ For example, UV‐responsive microcapsules were synthesized via Pickering emulsion polymerization using TiO_2_ and SiO_2_ nanoparticles as the Pickering agents. The microcapsules were embedded into waterborne polysiloxane, which was further fabricated with fully water‐based self‐repairing superhydrophobic coatings. When mechanical damage or contamination by organic was found in the coating, the coating can be recovered under UV light.^[^ ^4a^ ^]^ TiN nanoparticles with outstanding plasmonic properties are promising candidates for photothermal conversion (Figure 5c). Ma et al. developed a novel nanocontainer TiN@SiO_2_, which could rapidly absorb light energy and convert it into heat under NIR irradiation, thereby elevating the local temperature of the nanocontainer and ultimately promoting the release of the corrosion inhibitor.^[^ ^43^ ^]^ The study of nanocontainers that have the photothermal response could provide new insights for the development of intelligent protective coatings. Although photosensitive materials have been widely studied by researchers, their application in preparing light‐responsive MICs remains limited. In the future, more photosensitive materials can be used to design and prepare advance MICs to meet the needs of complex applications.

Photochromic molecules, such as azobenzene, ortho‐nitrobenzyl, and spiropyran‐based polymers, are commonly used to prepare intelligent light‐responsive microcapsules(Figure 5b).^[^ ^100^, ^112^ ^]^ For instance, Zhang's group developed reversible UV‐sensitive microcapsules by layer‐by‐layer self‐assembly (LbL) using azobenzene molecule, which could controllably switch between “on” and “off”. Under UV irradiation, the azobenzene molecule switched from a thermodynamically stable *trans* structure to a metastable *cis* structure, resulting in a change in the pore size of the microcapsules and the stable release of loaded drugs. Under visible light irradiation, the azobenzene molecule transformed into a *trans* structure from a *cis* structure, and drugs could be better encapsulated into microcapsules.^[^ ^112b^ ^]^ In summary, various photo‐sensitive materials and groups have numerous potential in preparing MICs. However, light stimulus still has some drawbacks, such as being susceptible to light scattering and biotoxicity.^[^ ^113^ ^]^ These problems may be solved by designing novel molecular structures to reduce light scattering and redshift the stimulus wavelength.^[^ ^114^ ^]^ Furthermore, more useful molecules could be designed by using artificial intelligence (AI). Therefore, advanced MICs with light response may need the collaboration of scientists in multiple fields, such as chemical, material, and computational scientists.

#### 4.1.2 Light Responsive Coating Matrixes

Light‐responsive materials have been used as coating matrixes to prepare light‐responsive MICs. Among them, SMPs have attracted intense interest because of their ability to change from a temporary shape to a memorized permanent shape upon external stimuli.^[^ ^115^ ^]^ SMP coatings incorporated with microcapsules have been prepared for self‐healing materials. As shown in Figure 5d, NIR light irradiation can convert light energy into thermal energy in situ, which activates the dissociation and reconstruction of the DA network to achieve self‐healing of shape memory coatings. For example, Yu et al. developed self‐healing waterborne PU coatings through the introduction of DA bonds into the polymer backbone. The addition of hybrid polydopamine and graphene oxide nanoparticles made coatings to achieve light‐triggered self‐healing.^[^ ^97^ ^]^ Wang et al. fabricated a novel NIR‐triggered self‐healing and anti‐corrosion coating by crosslinking maleimide end‐capped PU and furan‐modified CeO_2_@PDA via DA bonds. Under NIR irradiation, the coating exhibited significant corrosion resistance and self‐healing ability because of the thermally reversible crosslinked coatings and the excellent photothermal conversion ability of the PDA nanoparticles.^[^ ^101^ ^]^ Although MICs based on light‐responsive coating matrixes have been fabricated, more types of materials with light‐responsiveness need to be explored for MICs. Furthermore, multiple light‐responsive mechanisms can also be designed to achieve optimal application effectiveness.

In conclusion, light‐responsive MICs have broad application prospects owing to their high precision, remote control, and instantaneous “on” and “off” characteristics. However, there remains significant potential for innovation. In the future, efforts should be focused on improving the physical, chemical, mechanical, and surface properties of MICs to enhance their performance and broaden their applicability in the field of intelligent composite coatings.

### 4.2 Mechanical Response

Mechanical response is one of the easiest methods to trigger release, research on the release of the core materials triggered by mechanical force has gradually emerged.^[^ ^116^ ^]^ The earliest application of mechanically responsive microcapsules can be traced back to 1957.^[^ ^11a^ ^]^ Microcapsules dispersed in carbonless paper could be broken under the pressure of a pen and released dyes on the next page of paper to leave the mark. Subsequently, mechanical responsive microcapsules based on different force types were developed and used in self‐healing coatings,^[^ ^46^, ^117^ ^]^ flavor encapsulation,^[^ ^118^ ^]^ and drug delivery.^[^ ^119^ ^]^ In this section, we will introduce mechanical stimulus‐responsive microcapsules from four aspects: stretching, pressing, osmotic pressure, and friction (Figure 5e).

#### 4.2.1 Stretching

Stretchable materials such as films and fibers have been incorporated with diverse microcapsules. When the deformation of stretchable materials exceeds the bearing capacity of the internal microcapsules, it will lead to the rupture of microcapsules, resulting in the release of the core material. For instance, Lee's group designed mechanically‐activated microcapsules which were capable of strongly adhering to a fibrous matrix. The microcapsules could be ruptured upon uniaxial stretching of non‐woven fabric dressings, leading to a self‐regulated release of anti‐biotics.^[^ ^68b^ ^]^ In addition, Zhang et al. synthesized PU shape memory microcapsules and integrated them into stretchable polyvinyl alcohol (PVA) films. At a temperature slightly above the melting point of the PU shell polymer, stretching the PVA films could lead to the deformation of PU microcapsules. More importantly, this kind of deformation could recover after the dissolution of PVA in water.^[^ ^120^ ^]^ However, this kind of MICs is only suitable for flexible and deformable substrates, such as fabric, rather than fixed substrate such as glass or large equipment.

#### 4.2.2 Pressing

Pressure‐sensitive microcapsules can release core materials under applied pressure because of the deformation or rupture of microcapsules. For example, Rajamanickam et al. fabricated mechanical stimuli‐responsive hybrid hollow capsules through the LbL approach. The microcapsules exhibited pressure‐induced and controlled release of loaded drugs for multiple cycles.^[^ ^102a^ ^]^ Tae's group designed spherical elastic hollow microcapsules, which were further added into PDMS films. Under uniaxial mechanical stimulation, hydrophilic drugs could be released from these elastic hollow microcapsules.^[^ ^119^ ^]^ In summary, microcapsules can release internal substances quickly and accurately under pressure stimulation, so they have broad application prospects in biomedicine, intelligent anti‐corrosion coatings, and other fields.^[^ ^46^, ^119^ ^]^

#### 4.2.3 Osmotic Pressure

In a low osmotic pressure environment (such as low‐concentration aqueous solution or water), there is an osmotic pressure difference between the inside and outside of the microcapsule. When the maximum value is reached, the microcapsule ruptures and contents are released. For instance, Jeong et al. prepared three‐emulsion microcapsules by microfluidic technology and realized the on‐demand release of drugs in microcapsules.^[^ ^102b^ ^]^ The obtained microcapsules have two shells, one is an inner oil shell and the other is an outer hydrogel shell. Only when the oil shell ruptures, the smaller size of the drug was released; When both the oil shell and the hydrogel shell broke, both sizes of the drug were released. In addition, Weitz's group used microfluidic technology to prepare stimulus‐response microcapsules that can trigger the release of encapsulated cargo at a low rupture osmotic pressure.^[^ ^121^ ^]^

#### 4.2.4 Friction

Friction‐sensitive microcapsules are usually triggered by substrate wear. For instance, Li et al. prepared tung oil@PSF microcapsules by solvent evaporation method. After the microcapsules were broken during friction, tung oil was released to form a boundary transfer lubrication film, which had a lubricating effect on the epoxy coating.^[^ ^45^ ^]^ Luo's team prepared PAO oil@PS microcapsules and incorporated them into the epoxy resin matrices. The released PAO oil from the microcapsules during the friction process produced a boundary lubricating film, which could prevent the direct contact of two rubbing surfaces, and thus greatly improved the lubrication properties of the epoxy composites.^[^ ^122^ ^]^

Taken together, mechanical responsive microcapsules have a convenient release trigger mode and can release core materials rapidly. However, to improve the integrity of microcapsules when embedded into the coating, the selection of materials release effect, and compatibility with the coating, three development directions may be referenced: 1) design simpler and more efficient synthesis strategies, 2) optimize the mechanical responsive performance, and 3) develop advanced processing means. These directions may promote further development of mechanical stimulus response microcapsules.

### 4.3 Thermal Response

Temperature has become a fundamental and precisely controllable method for tuning material properties, leading to its wide use in developing advanced stimulus‐responsive systems.^[^ ^83^ ^]^ Different thermal‐responsive microcapsules (i.e., shell and core) and coating materials (i.e., matrixes) have been used to prepare diverse MICs depending on the phase transformation or volume change of these materials. In this section, we will introduce and discuss these materials and novel MICs in detail, as shown in Figure 5f‐h.

#### 4.3.1 Thermal Responsive Microcapsules

A typical thermally responsive microcapsule is prepared with a thermosensitive polymer as the capsule wall, and its release mechanism is based on the volume/phase change of the thermosensitive polymer (Figure 5f).^[^ ^123^ ^]^ For example, when the temperature rises above or reduces below the low critical solubility temperature (LCST), thermosensitive responsive microcapsules can change their size depending on the change in temperature because of the hydrophilic‐hydrophobic transition of thermally responsive polymers.^[^ ^123^, ^124^ ^]^ Thermo‐responsive microcapsules were prepared using poly(N, N‐diethylacrylamide) hydrogels as shell material, which showed volume‐phase transition around 34 °C. At low temperatures, the hydrogel shell could be highly swollen by water and provided high permeability.^[^ ^125^ ^]^ Deng's group modified emulsion droplets consisted of linalool and organosilica precursor with carbon‑carbon double bonds, followed by the precipitation‐polymerization with *N*‐Vinylcaprolactam (VCL) as monomers and *N*, *N*’‐Bis(acryloyl) cystamine as crosslinker, thereby obtaining dual‐responsive linalool microcapsules. When the temperature was higher than 32 °C (LCST = 32 °C), the strong hydrogen bond between the acrylamide group of the PVCL chain and the water molecule broke, resulting in the contraction of the polymer chain and further causing the release of the linalool.^[^ ^103^ ^]^ Compared with LCST polymers, polymers with an upper critical solution temperature (UCST) are insoluble below UCST but soluble above UCST. Recently, Tong's group has made significant progress in the development of novel biocompatible UCST polymers. The surface of HMSNs was grafted with UCST polymers, which were used to load the doxorubicin drug and the photothermal conversion agent indocyanine green. The presence of indocyanine green raised the temperature of the solution under NIR radiation, leading to the alteration of the chain conformation of the UCST polymer, eventually the drug was released from the microcapsule.^[^ ^126^ ^]^ In summary, the volume/phase change of the thermosensitive polymer is an attractive method to prepare the shell of microcapsules, exhibiting numerous potential in different applications such as anti‐bacterial operation in complex environments.

Another thermally responsive route is using PCMs as a shell material or core material. When the temperature rises above the inherent phase‐transition temperature, such as the glass transition temperature (*T*_g_), and melting temperature (*T*_m_), the shell of microcapsule expands and ruptures. For example, Ren et al. developed thermal‐responsive microcapsules with brittle‐ductile transition properties based on the heat‐releasing nature during cement hydration. Microcapsules could change from a stiff and glassy state to a soft and rubbery state when in wet conditions, which could reduce the breakage rate of microcapsules and improve the recovery rate.^[^ ^74d^ ^]^ Zhang's group designed Ce(NO_3_)_3_@ethylene vinyl acetate (EVA) microspheres. EVA microspheres could melt when the temperature was above *T*_m_ and release Ce(NO_3_)_3_ corrosion inhibitor, resulting in the inhibition of the corrosion activity inside the scratches of the coating.^[^ ^127^ ^]^ In addition, the thermal responsive PCMs as core materials mainly depend on the large amount of latent heat stored or released during the solid‐liquid phase change process (Figure 5g). For instance, Yuan et al. prepared paraffin@MUF PCMCs, and applied them to permanent room temperature vulcanized (PRTV) silicon rubber to prepare an anti‐icing coating on glass.^[^ ^18e^ ^]^ The PCMCs in the coating could absorb and release energy to the environment repeatedly at a specific temperature, exhibiting a “temperature switch” effect. Therefore, the PCMCs/PRTV coating has potential applications in the field of glass insulators under glaze ice. Gu et al. used *n*‐eicosane as the PCMs, melamine, urea, and formaldehyde (MUF) as the inner wall, and PANI as the outermost layer to prepare microcapsules with both temperature control and low infrared emissivity. After coating the microcapsules onto polyester fabrics, the fabrics can be cooled down by a maximum of 11.2 °C compared to the untreated fabrics, and the temperature control process could last for 27 min.^[^ ^47^ ^]^ Taken together, PCMCs that can effectively tune the temperature of the coating materials demonstrate huge possibilities in the preparation of thermal management materials such as infrared stealth materials. However, more kinds of PCMCs should be developed in the future.

#### 4.3.2 Thermal Responsive Matrixes

In addition to the temperature‐responsive microcapsules, SMP as an emerging thermo‐sensitive intelligent material, can also be used as a temperature‐responsive coating matrix. The mechanism for the shape recovery of SMP coating in response to thermal stimulation is that when the temperature rises above the *T*_g_, the state of SMP will change from a glassy state to a rubber state. The stored strain energy in the deformation area and the applied contracting force can bring the defect region spatially close (Figure 5h).^[^ ^43^ ^]^ According to this mechanism, microcapsules embedded with Alodine 5200 and heating agents were added to shape memory PU. The heat could be released by the exothermic reaction between the heating agent and oxygen, causing shape memory recovery of PU.^[^ ^128^ ^]^ Huang et al. used epoxy SMP to prepare a new self‐healing shape memory coating. When the heat was applied, the shape memory effect induced the thorough closure of the physical barrier of the coating. The coating exhibited excellent self‐healing properties.^[^ ^129^ ^]^ In summary, SMP is a kind of materials to prepare thermal‐responsive MICs. Compared with the other two kinds of thermal responsive MICs, SMP acts as a coating matrix and is independent of the microcapsules. Therefore, various microcapsules can also be loaded into SMP coating to prepare novel and multi‐functional MICs.

Compared to the mechanical response, the temperature response can enhance the triggering efficiency when placed in an extreme environment. This stimulus mean is also easy to operate, and the transition temperatures for different systems are adjustable, leading to their wide use in the controlled release of drugs, industrial catalysis, and hydrogel.^[^ ^130^ ^]^ However, the rigorous demand for material restricts further research on temperature‐responsive MICs. Therefore, it is necessary to design and develop more kinds of temperature‐sensitive materials and broaden their application ranges.

### 4.4 Magnetic Response

The magnetic field has been widely used to stimulus responsive microcapsules because of its inherent benefits such as wireless and remote control, ease of integration, high accuracy, and biocompatibility.^[^ ^131^ ^]^ There are two methods in which the magnetic responsive MICs can be achieved, one is the magnetothermal effect, and the other is the uniform distribution of microcapsules under magnetic field.

#### 4.4.1 Magnetothermal Responsive Materials

The mechanism of magnetothermal effect is that the magnetic material in MICs can be magnetized and heated by an external magnetic field, resulting in a raised temperature and further exhibiting magnetic response (Top of Figure 5i). For example, Studart's group designed a novel magnetic trigger release system. The core material generated heat under a magnetic field, causing the covered temperature‐sensitive microcapsule to rupture, and the microcapsule contents could be released within seconds. This technology has the potential for use in the on‐demand release of cement promoters.^[^ ^132^ ^]^ Similarly, Liu et al. prepared magnetothermal responsive composite submicron particles cobalt ferrite@PEG gel, for recyclable catalytic applications. Through the method of magnetic separation and magnetothermal response release, the loadings and carriers were recovered, and the recycling of loadings and carriers was realized.^[^ ^104^ ^]^

#### 4.4.2 Uniform Distribution Under Magnetic Field

Another magnetic response is the drive of microcapsules containing magnetic particles under the control of an external magnetic field (Bottom of Figure 5i).^[^ ^49^, ^133^ ^]^ For example, Wang et al. prepared magnetic microcapsules containing magnetic multi‐wall carbon nanotubes (MWCNTs) by in situ polymerization, in which BTA is a core and MWCNTs is a magnetic target. Magnetic microcapsules rapidly migrated in the coating solution because of the applied magnetic field, which shortened the migration route of BTA and accelerated the self‐healing of the coating.^[^ ^133b^ ^]^ Liu and his colleagues incorporated tung oil@calcium alginate microcapsules (with built‐in anti‐fouling particles of conductive polyaniline and magnetic particles of Fe_3_O_4_) into PU coating to prepare magnetically responsive self‐healing PU coatings. Under the action of a magnetic field, the magnetic particles can be uniformly distributed and thus uniformly distributed functional particles, which provided an effective idea to improve the performance of coatings.^[^ ^49^ ^]^ Long et al. embedded Fe_3_O_4_ particles into a polymer shell. The Fe_3_O_4_ particles aligned along the field direction and stretched the shell when the magnetic field was applied, resulting in the compression of the microcapsules and release of the core contents.^[^ ^133a^ ^]^ Furthermore, the shape could be restored after the magnetic field was removed. This approach provided the opportunity to accelerate the release of microcapsules in a controlled manner while maintaining the integrity of the shell. In conclusion, magnetic response meets the requirements for an environmentally friendly trigger and remote trigger.

Many advantages, such as quick response and strong penetration, can be found in magnetic response compared with other types of responses, especially for thermal response. However, there are still difficulties in accurately locating magnetic fields. Potential obstacles to the safety and practicality of equipment that generates and maintains magnetic fields have also limited its use in many fields, such as life, health, and energy. Therefore, more miniaturized, high‐resolution, high‐security, and low‐cost equipments should be developed.

### 4.5 pH Response

The development of pH‐responsive polymers has attracted tremendous interest over the past two decades.^[^ ^134^ ^]^ In general, pH‐responsive polymers can respond to the variation of environment pH through the degradation or conformational transformation of polymer molecules.^[^ ^135^ ^]^ In this section, we will introduce two mechanisms of microcapsules with pH‐responsive polymers as shells in detail.

#### 4.5.1 Polymer Degradation

Microcapsules can controllably delivery loadings after their polymeric shell degrades. Proper materials of shell that lack durability under acidic or alkaline conditions can cause them to disintegrate or degrade (Top of Figure 5j). For example, Qian et al. reported for the first time the use of PDA as a pH‐sensitive gatekeeper for MSNs. PDA shell could be degraded in an acidic environment, leading to the rapid release of BTA from MSNs.^[^ ^42^ ^]^ Similarly, porous PSF microcapsules coated with stearic acid and loaded with scale and corrosion inhibitor HDPE were prepared, which exhibited significant pH‐triggering activity in alkaline environments because of the reactivity of carboxylic acid group of stearic acid to alkaline stimuli.^[^ ^19b^ ^]^ Amart from these pH responsive materials, ZIF‐8 can be decomposed under weak acidic conditions due to the instability of the coordination bonds in ZIF‐8.^[^ ^72^ ^]^ For example, Huang et al. created BTA@ZIF‐8/SiO_2_ nanocontainers, which exhibited excellent pH‐responsive release behaviors because the hydrolysis of metal‐linker bonds in ZIF‐8 can be facilitated by protonation of the linker under acidic conditions.^[^ ^136^ ^]^ Yang et al. developed a pH‐responsive hydrophilic controlled release system BTA/tannic acid@ZIF‐8 for waterborne epoxy resin self‐healing anti‐corrosive coating. The inhibitors BTA and tannic acid were released from the system when corrosion occurred, thereby endowing the coating with anti‐corrosion and self‐healing ability.^[^ ^50^ ^]^ It should be mentioned that the degradation products from the shell of microcapsules need to be eco‐friendly and safe for many applications, such as life health, and electron device. Therefore, more pH‐sensitive materials are being developed in the future.

#### 4.5.2 Polymer Conformational Transformation

Another pH response mechanism is to cause conformation transformation of polymer molecular chains under pH change, as shown in the bottom of Figure 5k. Alginate and chitosan are natural pH‐responsive polymers with variations in solubility.^[^ ^134^, ^137^ ^]^ For example, Liu et al. developed a self‐sensing and active corrosion protection coating that incorporated pH‐sensitive 1,10‐phenanthrolin‐5‐amine (APhen)@chitosan/alginate‐covered CaCO_3_ microcontainers. The microcontainers could release APhen in a controlled fashion responding to pH variation, which made the coating demonstrate an active corrosion protection effect.^[^ ^137a^ ^]^ Cui's group prepared pH‐responsive microcapsules through a LbL method and added them to the epoxy resin coating. CS in the outermost layer of the microcapsules had an obvious stimulus response to acidic pH, so it had a stronger repair effect and corrosion resistance on the corrosion coating.^[^ ^137b^ ^]^ In addition to natural polymers, synthetic polymers can also undergo conformational transformations when pH changes. For example, Zhang's team successfully polymerized poly(2‐(Dimethylamino)ethyl methacrylate) (PDMAEMA) on the surface of mesoporous silica nanoparticles. Under neutral condition (pH 7), PDMAEMA was in a hydrophobic state, and the organic chain segment was in a folded state, covering the pores on the surface of mesoporous silica. While under acidic condition (pH < 7), the tertiary amine group in PDMAEMA reacted with the ionized hydrogen ions and formed ammonium bicarbonate, and PDMAEMA changed from hydrophobic to hydrophilic. Thus, the obstruction of the pores was reduced.^[^ ^105^ ^]^ Tavandashti et al. developed PANI hollow microspheres that are used for the encapsulation of organic corrosion inhibitor MBT. When exposed to pH > 7, the polymer chains were deprotonated. PANI shell shrinkage caused MBT molecules to be discharged through openings or cracks on the surface of the microsphere.^[^ ^138^ ^]^ Taken together, the conformation of natural and synthetic polymers can be affected and transformed triggered by the pH change. These polymers exhibit numerous potentials in fabricating pH‐responsive MICs.

In summary, tremendous progress has been made in designing various pH‐responsive microcapsules. However, their potential limitations or challenges are mainly reflected in the ranging of triggering pH values and uncontrolled release of contents under continuous pH change. To meet the needs of practical applications it is necessary to improve the molecular structure of materials to develop pH‐responsive materials with high performance.

### 4.6 Ion Response

Ion response is another method of chemical stimulus response, which is mainly activated by the interaction between shell materials of microcapsules and specific ions such as S^2‐^ and Cl^‐^.^[^ ^6^, ^35^ ^]^ According to different trigger ions, a variety of materials can be selected to prepare microcapsules for preparing ion‐responsive MICs. In this section, we focus on the ion response induced by the common ions of S^2‐^ or Cl^‐^/H^+^.

#### 4.6.1 S^2‐^ Ion Response

As a common condition, aggressively corrosive ions are regarded as triggering ion‐responsive microcapsules.^[^ ^139^ ^]^ Sulfate‐reducing bacteria (SRB) are regarded as a type of representative corrosive microorganism existing in marine environments, which can convert sulfate into S^2‐^ ion in their respiration. S^2‐^ ion can be regarded as a typical signal to accurately control the release of biocides (Top of Figure 5k). For example, Cai et al. designed a novel kind of ZIF‐8@MSN nanocontainers that respond to S^2‐^ ion. In marine environments containing SRB, the ZIF‐8@MSN nanocontainers can respond to S^2‐^ fluctuations caused by SRB corrosion. When the concentration of S^2‐^ ion is large, the ZIF‐8 dissolved and biocides were released in response.^[^ ^73^ ^]^ In addition, Chen et al. also fabricated BTA‐HMSN@ZIF‐8‐MNZ nanocontainers with typical S^2‐^ ion‐responsive characteristics. The release rate of the encapsulated MNZ and BTA rose with the increased sulfide‐ion concentrations, which greatly strengthened the anti‐microbial and corrosion inhibition performance of the nanocapsules.^[^ ^51^ ^]^

#### 4.6.2 Cl^‐^/H^+^ Ion Response

Apart from aggressively corrosive ions, cathodic and anodic reactions cause local pH changes in corrosive environments. When the materials are exposed to environments bearing Cl^‐^, Na^+^, H^+^, and OH^‐^ ions, these ions also result in the stimulus response of microcapsules (Bottom of Figure 5k).^[^ ^20^, ^77^, ^140^ ^]^ For example, Liang et al. synthesized Ca(OH)_2_ microcapsules with an ion‐responsive poly‐ionic liquids shell. By exchanging the anions of the PILs with aggressive anions (such as Cl^‐^ or SO_4_^2‐^), the hydrophobic PILs on the shell converted to hydrophilic. The Ca(OH)_2_ has relatively low water solubility, and it can gradually permeate outside the microcapsule through the shell.^[^ ^140a^ ^]^ Li's group fabricated ion response microcapsules via the LbL method. HNTs loaded with MBT were used as the core, while poly(diallyl dimethylammonium chloride) (PDDA) and poly (styrene sulfonate) (PSS) polyelectrolytes films used as the shell. Under the stimulation of Cl^‐^, H^+^, or OH^‐^ ions, there are different degrees of loaded inhibitor release.^[^ ^77d^ ^]^ In summary, similar to pH‐responsive microcapsules, ion‐responsive ones can be sensitively triggered under the gradual invasion of ions. Ion response can open new avenues and provide fresh direction for the controlled release of intelligent microcapsules in the future. However, many ion‐responsive coatings can only respond to specific ions, which greatly limits their application ranges. In addition, the response sensitivity should be considered and improved in the following studies.

In addition to the response types discussed above, electro‐responsive MICs begin to attract the attention of researchers. For instance, electrochromic microcapsules can change color due to electron transfer under electrical stimulation. When combined with textiles, wearable smart textiles can be prepared.^[^ ^141^ ^]^ Nevertheless, current research on electrically responsive MICs remains relatively limited, and future studies are expected to place greater emphasis on this field to explore its potential applications and underlying mechanisms.

### 4.7 Multiple Responses

In many cases, different environmental changes may occur at the same time, so a single stimulus response is not sufficient for practical applications.^[^ ^145^ ^]^ Therefore, having multiple stimulus‐responses at the same time is very advantageous. At present, multi‐stimulus responsive materials, particular those integrating complementary triggers (such as light/pH and thermal/pH as summarized in Table 2), have garnered increasing attention. Among them, the most common application in MICs is the combination of light and pH responses because of their spatiotemporal controllability and environmental compatibility. For example, Zhao et al. modified simvastatin@HMSNs with PDA and polycaprolactone diacrylate to prepare nanocontainers with pH and NIR responses. PDA has both pH and NIR sensitivity, so it can act as a “gatekeeper” to precisely control simvastatin leaching from the nanocontainers.^[^ ^142^ ^]^ Similarly, Guo et al. assembled the PDA on the BTA@MSN surface to give the nanoparticles pH responsive and NIR triggered properties.^[^ ^53^ ^]^ In addition, Cong et al. also synthesized pH and UV dual‐responsive microcapsules by UV‐initiated polymerization of Pickering emulsions stabilized with SiO_2_ and TiO_2_ nanoparticles.^[^ ^143^ ^]^ In addition, You's group prepared ZnO@mesoporous PDA microspheres. The pH and NIR response characteristics of PDA and the photocatalytic activity of ZnO nanoparticles made the microspheres exhibit excellent multi‐responsive properties under UV/NIR/acid/base conditions (Figure 5l).^[^ ^106^ ^]^ Taken together, the multiple responses can respond to several triggering factors to deal with different environmental changes, which exhibits more intelligence and flexibility. Therefore, the integration of multiple responses may endow the materials with wider applications in both industrial and daily life.

The above‐mentioned stimulus‐response modalities are directly related to the prospective applications of MICs. In this section, the mechanism of those stimulus responses was discussed. Due to the advances in material design and manufacturing technologies, the sophistication of MICs has increased, and some new application scenarios have been raised. In the following section, we will go into great depth about the applications of MICs according to their functions, including anti‐corrosion, anti‐fouling, self‐lubricating, and temperature regulation.

## 5 Applications of MICs

Conventional coatings have been used in various fields around the world. However, they exhibit several problems such as single functionality, non‐intelligent responsiveness, and short lifespan, limiting their further development.^[^ ^146^ ^]^ In contrast, MICs can exhibit numerous functions in complex applications because of the existence of microcapsules. Given the huge development potential of MICs, this section classifies and discusses the emerging applications of MICs according to their functions, including anti‐corrosion, anti‐fouling, self‐lubricating, and temperature regulation.

### 5.1 Anti‐Corrosion

Corrosion has a direct impact on the service life, reliability, and safety of materials, and thus leads to the consumption of various resources and energy.^[^ ^147^ ^]^ Therefore, corrosion protection has been a crucial issue in diverse fields such as energy, chemicals, power, and transportation. MICs can act as a passive barrier to protect the underlying metal from corrosion by hindering detrimental ionic pathway and electrolyte permeation. These intelligent coatings can be applied onto the surface through diverse methods such as spraying,^[^ ^20^ ^]^ scraping,^[^ ^19^, ^20^, ^21^ ^]^ spinning,^[^ ^20^, ^22^ ^]^ brushing,^[^ ^23^ ^]^ and dipping.^[^ ^18^, ^20^, ^24^ ^]^ By virtue of such application methods, applying MICs to the surface of materials is a widespread and effective way to delay the arrival of corrosion, and therefore, extending the lifetime of materials.^[^ ^147b^ ^]^

#### 5.1.1 Basic Corrosion Protection

**Figure 6:**
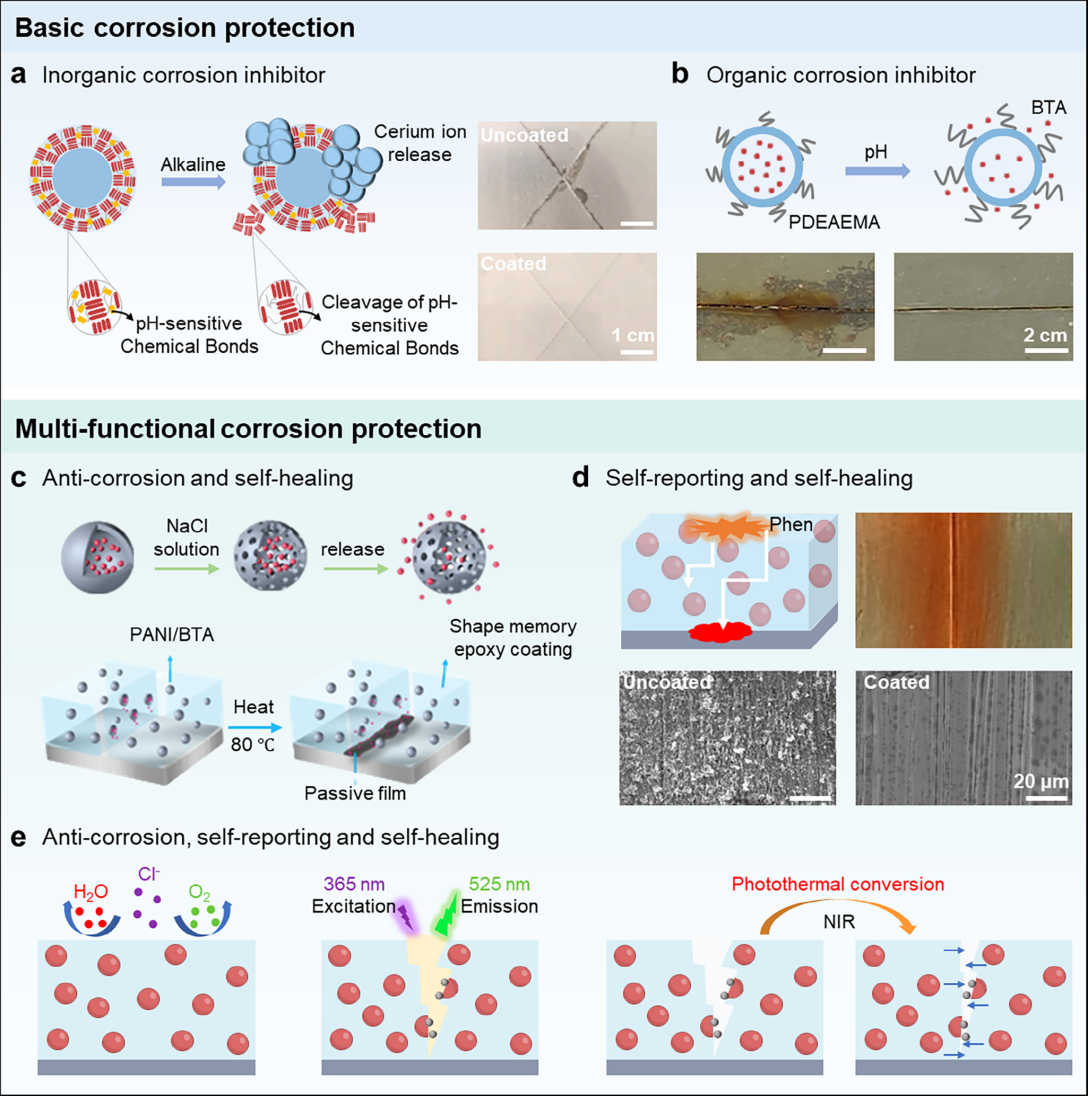
Anti‐corrosive MICs. a) Anti‐corrosive performance of MICs with inorganic corrosive inhibitor‐loaded microcapsules. Reproduced with permission.^[^ ^74b^ ^]^ Copyright 2021, Elsevier Ltd. b) Anti‐corrosive performance of MICs without and with BTA. Reproduced with permission.^[^ ^23^ ^]^ Copyright 2022, Elsevier Ltd. c) Anti‐corrosion and self‐healing of shape memory MICs based on BTA@PANI microcapsules. Reproduced with permission.^[^ ^52^ ^]^ Copyright 2022, Elsevier Ltd. d) Anti‐corrosion and self‐reporting of MICs using Phen@MSN/CS. Reproduced with permission.^[^ ^82^ ^]^ Copyright 2020, Royal Society of Chemistry. e) Anti‐corrosion, self‐reporting, and self‐healing performance of shape memory MICs based on 8‐HQ@PANI. Reproduced with permission.^[^ ^19c^ ^]^ Copyright 2024, American Chemical Society.

Different corrosion inhibitors, including inorganic and organic, have been encapsulated into the microcapsules in MICs, which can achieve anti‐corrosion under external stimulus. For instance, pH‐responsive cerium nitrate@melamine formaldehyde‐epoxy was prepared by Sarabi's group. Inorganic cerium nitrate can be released in an alkaline environment and yield corrosion protective layers of cerium oxide/hydroxide by reacting with corroding metal sites (**Figure** **6**a).^[^ ^74b^ ^]^ Organic corrosion inhibitors with sulfur, oxygen, or nitrogen heterocyclic structures such as 8‐HQ,^[^ ^21^, ^24^, ^67^, ^78^ ^]^ MBT,^[^ ^77a–c^ ^]^ and BTA^[^ ^42^, ^76^ ^]^ are also commonly used to form anti‐corrosive thin films through electrostatic interactions or coordination complexes.^[^ ^147b^ ^]^ For example, the encapsulated BTA in BTA@MSNs‐PDA coating was trapped inside the MSNs under a neutral pH condition. When being in an acidic environment, BTA@MSNs‐PDA coating could release BTA quickly, thus showing the superior anti‐corrosive effect on the damaged areas.^[^ ^42^ ^]^ MSN‐BTA@PDEAEMA microcapsules were prepared and used to compare the anti‐corrosive effect with pure epoxy coatings. Researchers found that the former exhibited excellent anti‐corrosive effects because of the controlled release of BTA (Figure 6b).^[^ ^23^ ^]^ In conclusion, diverse corrosion inhibitors have been used to prepare MICs, which have shown enormous potential in corrosion protection. However, more means to trigger the release of corrosion inhibitors from MICs should be developed because of the complexity and dynamics of the actual application environment.

#### 5.1.2 Multi‐Functional Corrosion Protection

In addition to the basic protection of corrosion inhibitors, the realization of multi‐functions and long‐term protection (e.g., active corrosion protection, corrosion sensing, and self‐healing) in one coating has attracted the attention of more and more researchers and industrial people.^[^ ^148^ ^]^ In recent years, Zhang's group has produced a series of multi‐functional anti‐corrosion coatings by combining microcapsules and SMP coatings, such as BTA@PANI, TiN/BTA@SiO_2_, or 8‐HQ@PCL microcapsules incorporate into epoxy resin.^[^ ^43^, ^52^, ^129^ ^]^ For example, PANI could realize the controllable release of BTA through ion response, achieving an anti‐corrosive effect. More importantly, the thermo‐responsive SMP epoxy coating could reduce the scratch size after heating, which showed a positive effect on the self‐healing of the coating (Figure 6c).^[^ ^52^ ^]^ Wang's group prepared MICs with autonomous warning and repairing effects. 1,10‐phenanthroline (Phen) loaded in the microcapsule can quickly detect Fe^2+^ ions generated after corrosion and can form a dense film on the surface of the steel to prevent the substrate from corrosion. As shown in Figure 6d, the steel coated with neat epoxy is severely corroded with numerous loose, while the corrosion of steel coated with Phen inhibitor coating is greatly inhibited.^[^ ^82^ ^]^ Liu's group prepared microcapsules loaded with IPDI for anti‐corrosion in deep‐sea environments. High hydrostatic pressure promoted the rupture of microcapsules, released IPDI, and facilitated the self‐healing of materials.^[^ ^149^ ^]^ In addition, three synergetic effects of corrosion inhibition, corrosion sensing, and photothermal self‐healing abilities simultaneously in one SMP MIC based on 8‐HQ@PANI have been achieved by Luo and her coworkers (Figure 6e). 8‐HQ could chelate with Al^3+^ ions to form 8‐HQ‐Al^3+^ coordinates with a conspicuous fluorescence.^[^ ^19c^ ^]^ The passivation and photothermal effect of PANI microcapsules could endow the coating with both corrosion inhibition and self‐healing properties. Overall, MICs used for multi‐functional anti‐corrosive applications have made great progress. However, there are still some drawbacks, such as large‐scale preparation, low‐coat, and universality for anti‐corrosive MICs, which need to be further explored. Researchers should consider the above factors when preparing anti‐corrosive MICs, which are important for industrial transformation.

### 5.2 Anti‐Fouling

Diverse pollutants such as bacteria, scale, and ice have caused serious problems in human health and industrial activity.^[^ ^151^ ^]^ Therefore, coatings with anti‐fouling characteristics have been extensively studied. For MICs in this review, they can play a crucial role in preventing the adhesion or removing the adhesion under the stimulation of bacteria, scale, and ice, making them a primary strategy for combating fouling.^[^ ^152^ ^]^ By fabricating microcapsules loaded with anti‐foulants, scale inhibitors, and PCMs, MICs with improved anti‐fouling repellency have been achieved. Due to their excellent performance, intelligent anti‐fouling coatings have notable applications in the industrial and medical fields, as discussed in the following section.

#### 5.2.1 Anti‐Bacteria

**Figure 7:**
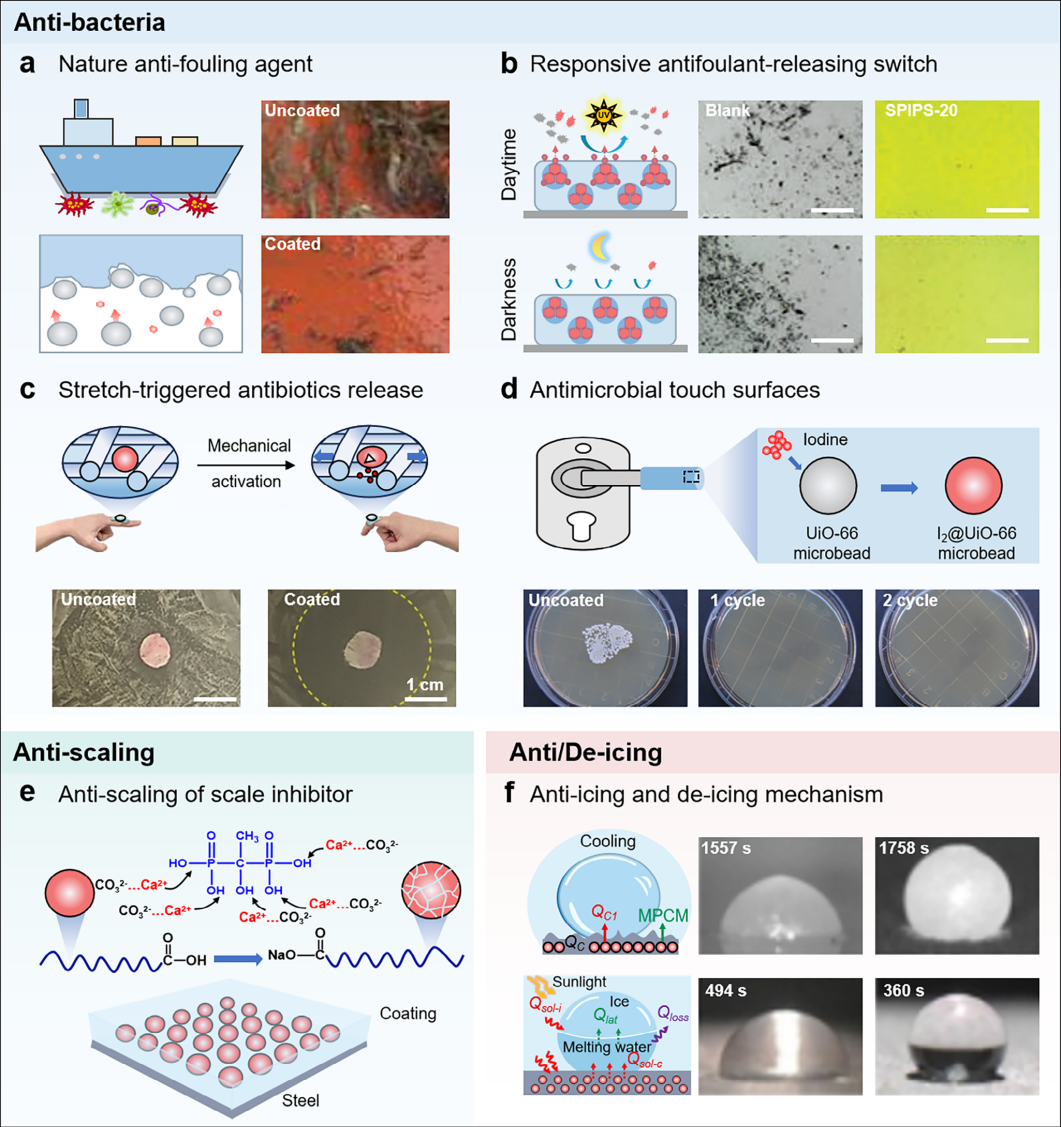
Anti‐fouling of MICs, including anti‐bacteria, anti‐scaling, and anti/de‐icing functions. a) Nature anti‐fouling agent capsaicin@PUF microcapsules for marine anti‐bacteria. Reproduced with permission.^[^ ^65a^ ^]^ Copyright 2020, Multidisciplinary Digital Publishing Institute. b) Responsive switch from “Defensive” mode in the daytime to “Offensive” mode in the darkness for anti‐organisms fouling of marine. Reproduced with permission.^[^ ^87^ ^]^ Copyright 2023, Wiley‐VCH. c) Mechano‐activated antibiotics release from CIF@PDA microcapsules for Anti‐Escherichia coli (E. coli). Reproduced with permission.^[^ ^68b^ ^]^ Copyright 2021, Royal Society of Chemistry. d) Anti‐microbial touch surfaces formed by PU polymer with UiO‐66 microparticles. Reproduced with permission.^[^ ^5^ ^]^ Copyright 2024, Wiley‐VCH. e) Anti‐scaling mechanism of coatings with pH response microcapsules. Reproduced with permission.^[^ ^19b^ ^]^ Copyright 2022, Elsevier Ltd. f) Anti/de‐icing mechanism of coatings with PCMCs. Reproduced with permission.^[^ ^150^ ^]^ Copyright 2024, Elsevier Ltd. Copyright 2023, Wiley‐VCH.

Biofouling (e.g., undesirable biomolecules, microorganisms, and bacteria) growing in marine vessels always leads to high fuel consumption and excessive maintenance costs.^[^ ^8^ ^]^ Currently, the incorporation of anti‐fouling agents into MICs is the most economical and effective means to combat marine biofouling, and research in this field is developing rapidly.^[^ ^153^ ^]^ For example, a form of environment‐friendly silicone oil/capsaicin@PUF microcapsules were prepared, which were further introduced into zinc acrylate resin to obtain bioinspired anti‐fouling coatings. As shown in **Figure** **7**a, the panels coated with anti‐fouling coatings showed almost no marine life compared with the uncoated panels, which were covered with widespread biofouling.^[^ ^65a^ ^]^ Inspired by the skin of poison dart frogs, Zhang's group designed a MIC with “offensive” and “defensive” modes that can reactively adjust the release of anti‐fouling agents. As shown in Figure 7b, the modified TraNBA@ZIF in the slippery porous‐liquid‐infused porous surfaces (SPIPS) can decompose and release Tra in response to sunlight's UV radiation, and the coating can transform into a low‐SE silicone coating to meet the reduced nighttime anti‐fouling requirements. The results exhibited that the SPIPS infused 20 wt.% silicone oil showed excellent anti‐fouling performance with no algae attached either day or night.^[^ ^87^ ^]^ In summary, anti‐fouling agents loaded MICs exhibit excellent anti‐biofouling, which can be applied on the surface of many equipment for preventing the adhesion of biofouling from marine.

In addition to biofouling in marine, the attachment and growth of bacteria on the surface of medical devices can also lead to a serious public health crisis and worldwide panic.^[^ ^154^ ^]^ To address the clinical diseases caused by a bacterial infection in medical facilities, MICs have drawn increasing attention due to their promising capabilities to prevent bacterial growth.^[^ ^155^ ^]^ For example, a smart wound dressing for controlling bacterial infection was prepared by mixing PDA‐coated mechanically‐activated microcapsules in a non‐woven fabric. Without mechanical activation, CIF@PDA microcapsules exhibited negligible inhibition of bacterial growth, while significant inhibition zones were observed around gauze with CIF@PDA microcapsules activated by 50% strain of stretching (Figure 7c).^[^ ^68b^ ^]^ Maspoch's group incorporated iodine@UiO‐66 microparticles into a potentially biodegradable PU polymer to prepare anti‐microbial door handle covers to prevent cross‐contamination. The results showed that the door handle covers could be able to completely prevent the growth and transmission of S. aureus after multiple contamination (Figure 7d).^[^ ^5^ ^]^ Additionally, An's group developed an antimicrobial coating containing microspheres loaded with N‐butyl‐1,2‐benzisothiazolin‐3‐one. Applying this coating to doorknobs, a common site for pathogen transmission, researchers found that the load of microbial on handles treated with the antibacterial coating was also significantly reduced.^[^ ^156^ ^]^ Additionally, bioinspired antimicrobial coatings were prepared by co‐polymerizing phenolic derivatives with amino‐terminated ligands.^[^ ^157^ ^]^ These coatings could be applied to various woven and non‐woven‐based materials, demonstrating many advantages such as long‐lasting, biocompatibility, scalability, and eco‐friendly. Taken together, MICs loaded with diverse matters such as anti‐fouling and anti‐bacterial agents have been successfully prepared and used in anti‐fouling of biomedical fields. One question is that the biocompatibility of MIC materials needs to meet the requirements, and this requires the joint efforts of material scientists and medical scientists.

In summary, MICs can significantly improve anti‐fouling effectiveness and extend service life. Therefore, developing multi‐functional MICs is crucial to protect marine equipment and prevent infectious diseases. With future research, innovation, and collaboration, these coatings could usher in a new era of environmentally responsible fouling prevention in the marine and medical industries.^[^ ^152^ ^]^

#### 5.2.2 Anti‐Scaling

Scale deposition, especially in the petroleum industry, is a serious issue because of its potential safety hazards and huge economic cost.^[^ ^158^ ^]^ Developing anti‐scaling coatings appears to be an ideal and promising strategy to overcome the scaling problem.^[^ ^40^ ^]^ For example, Meng's group fabricated a scalable and robust bioinspired organogel coating by the combination of organogel materials and nanoparticles for sustainable scale resistance.^[^ ^158a^ ^]^ Wang's group synthesized a thermoplastic PU/polyvinylidene fluoride/microcapsule/carbon nanofiber composite coating using microcapsules loaded with EDTA‐Zn metal ion chelators. EDTA‐Zn was released through the slow release of microcapsules, which impeded the growth of CaCO_3_ crystals on the coating surface.^[^ ^20c^ ^]^ In addition, HEDP was incorporated into porous PSF microcapsules and coated stearic acid on the surface of the HEDP@PSF intermediate to synthesize pH‐responsive smart microcapsules. In addition, a pH‐responsive HEDP@PSF/SA‐PU coating was also prepared. With the dissolution of stearic acid under different pH conditions, HEDP was gradually released to the coating surface, and a stable intermediate HEDP:Ca^2+^ was formed. Therefore, pH‐responsive smart coatings exhibited long‐lasting anti‐scaling capabilities in alkaline environments (Figure 7e).^[^ ^19b^ ^]^ In conclusion, the efficient MICs used for anti‐scaling face challenges and development prospects, and continuous innovation is required to find a new avenue to alleviate energy shortage and support the double carbon targets.

#### 5.2.3 Anti/De‐Icing

Ice accumulating on various surfaces, such as aircraft wings, power lines, and wind turbine blades, can cause disastrous consequences and economic losses.^[^ ^29^, ^159^ ^]^ Hence, the prevention of icing for industrial facilities is essential and necessary. Photothermal anti‐/de‐icing materials have attracted significant attention due to their potential to address surface ice formation.^[^ ^150^, ^160^ ^]^ Photothermal anti‐icing can prevent or delay the icing behavior of the substrate surface through the photothermal conversion of the material. The delayed icing mechanisms are shown in Figure 7f. When the temperature decreases to the phase change temperature, the PCMCs inside the coating begin to undergo phase transition, solidification, and release latent heat (*Q*_c_). The heat transmission from the coating to the liquid droplet is *Q*_c1_. Owing to the transmitted *Q*_c1_ to the droplet, the cooling of the droplet will be delayed.^[^ ^150a^ ^]^ Photothermal deicing is to remove the ice covered on the surface of the object by the photothermal effect.^[^ ^150b^ ^]^ The energy balance of the melting ice can be given by: Δ*Q* = *Q*_sol‐c_ + *Q*_sol‐i_ ‐*Q*_loss_. *Q*_sol‐c_ is the main source of heat, which depends on the surface's photothermal conversion ability. When *Q*_sol‐c_ is large enough to make Δ*Q* > 0 (Δ*Q* is used for the latent heat required for ice melting, *Q*_lat_) the ice melts.^[^ ^88b^ ^]^ Based on the above mechanisms, Ren et al. synthesized PCMCs and fabricated a TiN@PCMCs‐PDMS coating, which could extend the freezing time of droplets by 201 s and shorten the melting time of ice by 134 s (Figure 6f).^[^ ^150a^ ^]^ Meng and his coworkers prepared a bio‐inspired anti‐icing material (BAM) bearing a PCMCs layer and a superhydrophobic photothermal (SPT) layer. The PCMCs layer can store energy in the daytime and release heat energy at night. The SPT layer displays excellent solar‐to‐heat conversion. Therefore, the BAM could achieve sustainable ice repellency.^[^ ^27b^ ^]^ To improve the photothermal conversion efficiency of coatings, Chu's group prepared *n*‐eicosane@TiO_2_/CuS PCMCs. These PCMCs could achieve efficient full‐spectrum absorption and exhibit significant superhydrophobicity due to the 3D platelet‐like architecture. Therefore, the multi‐functional hydrophobic coating loaded with PCMCs demonstrated excellent anti‐/deicing performance in extreme conditions such as freezing rain.^[^ ^161^ ^]^ Luo's group fabricated an effective anti‐icing/de‐icing coating based on multi‐functional butyl stearate@PANI microcapsules.^[^ ^162^ ^]^ Benefiting from the heat storage capacity of butyl stearate in the microcapsules, the coating could achieve all‐weather anti‐icing capability. Taken together, the integration of photothermal effect, phase‐change storage capability, and hydrophobicity opens an avenue for realistic long‐term anti/de‐icing applications.

### 5.3 Self‐Lubricating

**Figure 8:**
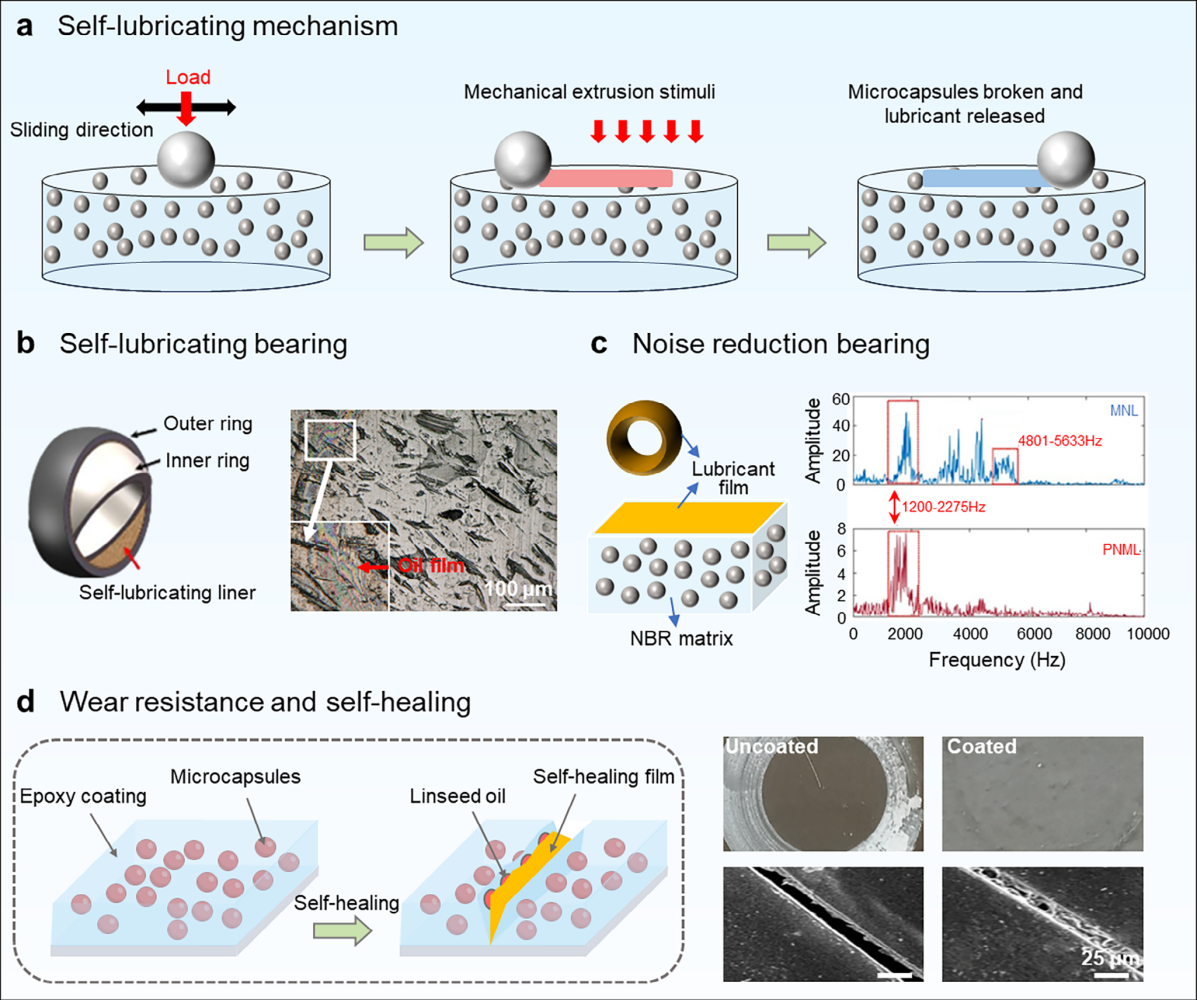
Self‐lubricating property of MICs. a) Self‐lubricating mechanism of MICs. Reproduced with permission.^[^ ^68a^ ^]^ Copyright 2021, Elsevier Ltd. b) Self‐lubricating bearing. Reproduced with permission.^[^ ^28d^ ^]^ Copyright 2022, American Chemical Society. c) Noise reduction bearing achieved by the addition of microcapsules. Reproduced with permission.^[^ ^166^ ^]^ Copyright 2023, Elsevier Ltd. d) Wear resistance and self‐healing of MICs. Reproduced with permission.^[^ ^167^ ^]^ Copyright 2022, Elsevier Ltd.

MICs with tailorable tribological and mechanical properties have been widely employed on mechanical parts to reduce friction and wear, which improves equipment performance and service life.^[^ ^163^ ^]^ Microcapsule technology can achieve the combination of solid lubrication and liquid lubrication while solving the limitations of storage and replenishment of liquid lubricants.^[^ ^164^ ^]^ Therefore, the fabrication of MICs for self‐lubricating by incorporating lubricant‐loaded microcapsules into a matrix has been widely reported.^[^ ^45^ ^]^ The self‐lubricating mechanism of MICs is shown in **Figure** **8**a. Under the stimuli of stress and friction, the shell of microcapsules breaks, and the lubricant encapsulated in microcapsules leaks into the wear surface, forming a lubricant film in the process of friction.^[^ ^68^, ^164^, ^165^ ^]^ According to the above mechanism, Zhang's group synthesized Oil@HMCNs‐epoxy resin composites as an adhesive for spherical plain bearings (Figure 8b). When rubbing on composites, an obvious oil film was observed on the surface, which exhibited a reliable self‐lubricating property.^[^ ^28d^ ^]^ Liu et al. designed a self‐lubricating bearing material with nitrile butadiene rubber and liquid nitrile butadiene rubber as the co‐matrix and microcapsules as the additive, which has excellent friction noise elimination ability. Figure 8c indicates that the addition of microcapsules into the co‐matrix greatly decreased spectral line and broadband noise in the range of 3300–5700 Hz compared with the pure co‐matrix.^[^ ^166^ ^]^ Wang's group prepared corrosion‐ and wear‐resistant difunctional coatings. Linseed oil released from microcapsules, which could fill the crack and protect the metal from corrosion. As shown in Figure 8d, a clear crack area was visible for the pure epoxy coating, while the scratched crack area was healed for the microcapsule coating. Linseed oil released from microcapsules also narrowed the wear tracks and reduced the wear debris significantly.^[^ ^167^ ^]^ Besides, Zhou's group developed a lubricant self‐pumping hydrogel inspired by the durable lubricity feature of earthworm epidermis. Glandular pockets storing lubricants in the hydrogel release the lubricant under high contact pressure conditions for ultra‐low friction and ultra‐long life.^[^ ^32c^ ^]^ Taken together, the findings expanded the application scopes of self‐lubricating MICs, providing ideas for the design of excellent wear‐resistant materials with engineering application prospects.

### 5.4 Temperature Regulation

**Figure 9:**
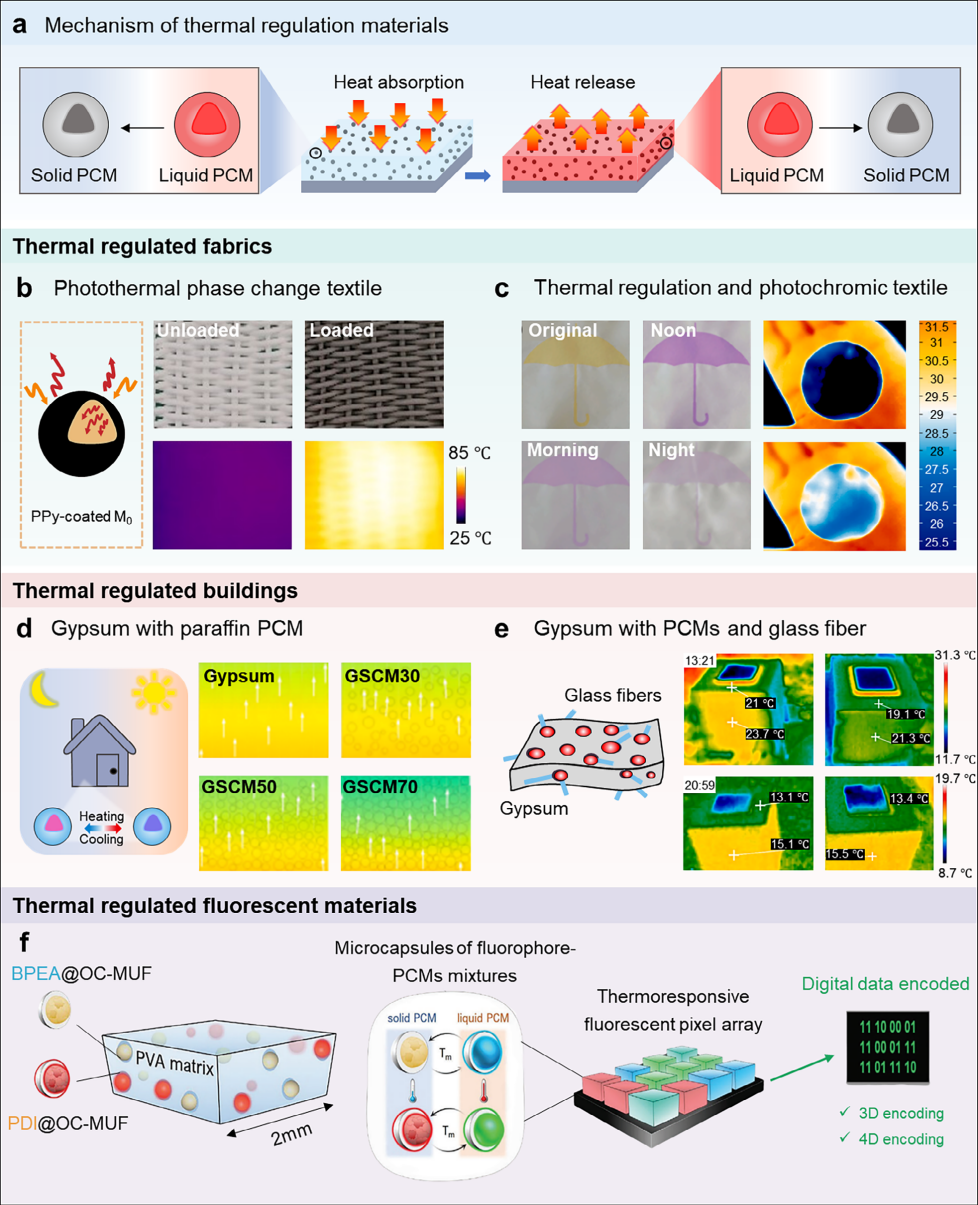
Temperature regulation of MICs. a) Mechanisms of heat absorption and release in temperature‐regulating materials. Reproduced with permission.^[^ ^168^ ^]^ Copyright 2021, American Chemical Society. b) Photothermal phase change textile. Reproduced with permission.^[^ ^89a^ ^]^ Copyright 2021, Elsevier Ltd. c) Thermal regulation and photochromic textile. Reproduced with permission.^[^ ^169^ ^]^ Copyright 2022, Elsevier Ltd. d) Temperature distribution images of gypsum with paraffin PCMCs. Reproduced with permission.^[^ ^91a^ ^]^ Copyright 2024, Elsevier Ltd. e) Thermal camera images of the test room with PCMCs‐gypsum plasterboard and the reference room. Reproduced with permission.^[^ ^170^ ^]^ Copyright 2022, Elsevier Ltd. f) Thermal‐regulated fluorescent materials. Reproduced with permission.^[^ ^171^ ^]^ Copyright 2024, Wiley‐VCH.

PCMs can absorb thermal energy as phase change takes place during the heating process, while releasing energy to the environment during a reverse cooling process (**Figure** **9**a). Therefore, PCMs have attracted lots of attention as potential materials for heat energy storage.^[^ ^168^, ^172^ ^]^ To effectively reduce the leakage of PCMs during the solid‐liquid phase transition and avoid the reaction of PCMs with the surrounding environment, microencapsulation of the PCMs to form PCMCs has been widely used in recent years.^[^ ^172^, ^173^ ^]^ Furthermore, PCMCs can be incorporated with many materials that are commonly used in thermoregulated smart fabrics and energy‐efficient buildings.^[^ ^174^ ^]^ Furthermore, various fluorophores have been encapsulated into PCMs to form multicolor thermoresponsive fluorescent materials, which could be used in sensing and anti‐counterfeiting.^[^ ^171^, ^175^ ^]^

#### 5.4.1 Thermal Regulated Fabrics

PCMCs integrated into various fiber and fabric materials have been extensively investigated by researchers.^[^ ^176^ ^]^ For example, Hu et al. prepared photothermal PCMCs modified with polypyrrole (PPy), which was further incorporated into PU matrix and fabricated a flexible photothermal phase change textile. Microcapsules coated with PPy can directly transfer the harvest heat into the PCMCs for energy storage. Therefore, the fabric containing PPy has significantly higher photothermal performance than the control samples under an irradiation intensity of 1 kW m^−2^ (Figure 9b).^[^ ^89a^ ^]^ Li's group integrated reversible photochromic PCMCs into cotton fabric and prepared photochromic and thermoregulated cotton fabric, which exhibited energy storage capacity and excellent photochromic performance.^[^ ^89b^ ^]^ Wang's group developed PCMCs containing eicosane and covalently graft them onto cotton fibers, obtaining functional textiles with long‐lasting heat regulation capabilities.^[^ ^28b^ ^]^ Additionally, Chen et al. prepared strawberry CS composite microcapsules by grafting photochromic nanocapsules onto CS microcapsules.^[^ ^169^ ^]^ These microcapsules could be applied in the fabric, which has good photochromic and thermal insulation performances. As shown in Figure 9c, the color of the printed fabric changed from yellow to deep purple under sunlight irradiation at noon, while the color of the printed fabric was darker than the color of the fabric in the morning or afternoon. Furthermore, when two pieces of fabrics (i.e., coated and control fabric) were placed on a hand, it was found that the surface temperature of the control fabric was higher than the coated fabric. In summary, with further research, fabrics based on PCMCs offer promise to meet the emerging needs of novel multifunctional fabrics.

#### 5.4.2 Thermal Regulated Buildings

Combining PCMCs with building materials is a promising composite building material that can minimize energy consumption inside buildings.^[^ ^154^, ^177^ ^]^ Excellent temperature regulation and flame retardancy are the keys to energy conservation of PCMCs building material.^[^ ^178^ ^]^ For example, superhydrophobic coatings were prepared by embedding thermochromic microcapsules into porous structures. The coating ensures radiative cooling performance at high temperatures and automatically switches to heating modes via solar absorption at low temperatures, thus exhibiting self‐switching optical properties.^[^ ^179^ ^]^ Zhao's group prepared PCMCs with single or multiple nuclei. They also prepared the composite blocks of gypsum with mononuclear or multinuclear PCMCs and investigated their temperature regulation. The results showed that the higher the content of mononuclear PCMCs, the slower the rate of temperature increase (Figure 9d).^[^ ^91a^ ^]^ Gencel et al. prepared a novel gypsum plasterboard that contained glass fibers PCMCs. The results showed that the room with PCMCs‐gypsum plasterboard showed a cooler outer surface temperature at the peak solar flux hours while a warmer temperature when the weather was cold (Figure 9e). Therefore, the PCMCs‐gypsum composites can be considered as an energy‐saving and indoor temperature regulating material in buildings.^[^ ^170^ ^]^ They also fabricated a novel PCMCs integrated glass fiber reinforced gypsum composite with sufficient light‐transmitting properties and thermal energy storage capacity.^[^ ^180^ ^]^ In addition, MICs loaded with PCMCs have also been used to prepare thermoresponsive smart windows.^[^ ^181^ ^]^ Paraffin nanoparticles in MICs showed high visible and light transparency when they were in the solid state, which was lost after melting, leading in opacity of MICs. Therefore, MICs integrating thermal and photothermal responses maintained high solar transmittance during cold conditions while significantly attenuating solar heat gain indoors under intense sunlight exposure.^[^ ^181^ ^]^ In conclusion, PCMCs in MICs can reduce building energy consumption and enhance environmental protection, which has broad application prospects in residential construction, thermal energy storage, solar energy collection systems, and thermal regulation.

#### 5.4.3 Thermal Regulated Materials

Thermal regulated materials such as thermochromic materials show enormous potential in various fields such as optical thermometers, diagnostic tools, photoelectronic devices, and anti‐counterfeiting labels. PCMCs encapsulated with different dyes have been widely studied and used for thermochromic materials. Roscini and Ruiz‐Molina group have developed a series of thermochromic materials based on PCMCs.^[^ ^171^, ^175^ ^]^ For example, paraffin‐fluorophore mixtures were encapsulated with melamine‐urea‐formaldehyde, which were further dispersed into poly(vinyl alcohol) film (PVC) to form thermoresponsive fluorescent pixels, as shown in Figure 9f.^[^ ^171^ ^]^ The integration of PCMCs incorporating diverse fluorophores and paraffin within individual pixels could enable highly dynamic multicolor emission, endowing the resulting arrays with dual capabilities: high‐security three‐dimensional data encoding and four‐dimensional storage capacity. In addition, diverse negative thermochromic responses across different spectral bands with varying shift amplitudes have been achieved by using a single dye, ketocyanines, which shows strong color sensitivity to various factors such as polarity, aggregation state, and pH.^[^ ^175b^ ^]^ The absorption characteristics could be tuned into the near‐infrared range through methine unit elongation, which facilitated the crucial colorless‐to‐colored transition mediated by negative thermochromism. In summary, by further optimizing the structure of dyes and the interface characteristics of microcapsules through molecular engineering, it is expected to achieve more precise temperature response threshold regulation and a wider spectral regulation range. With the deep integration of intelligent materials and internet of things technology, these multifunctional thermochromic systems will open up new application scenarios in real‐time biomedical monitoring and dynamic optical encryption.

Upon thorough examination of the existing research, it is evident that MICs are a popular choice for anti‐corrosion, anti‐fouling, self‐lubricating, and temperature regulation materials. In addition, MICs have also been used in other fields such as drug delivery and smart electronics. For example, various micro‐/nano‐capsules encapsulated with functional loads were prepared and used for tumor‐targeting drug delivery, anti‐inflammatory imaging and therapy, thermal monitoring of cell metabolism.^[^ ^64^, ^182^ ^]^ However, it is still a great challenge to achieve intelligence, durability, and multifunctional during the development process of MICs. Therefore, there is a pressing necessity to intensify efforts in optimizing MICs, aiming to improve material properties while being multifunctional and intelligent. The simultaneous presence of challenges and opportunities presents an intriguing outlook for the future of MICs.

## 6 Conclusion and Perspectives

**Figure 10:**
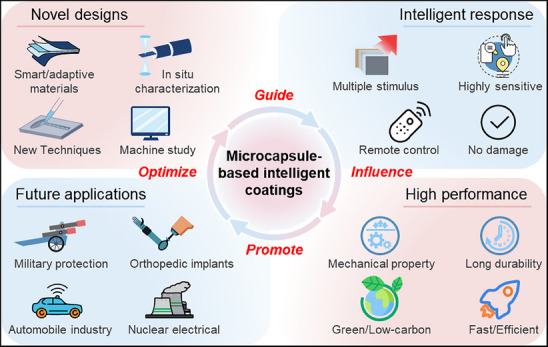
Schematic illustration of the future development perspectives of MICs from four aspects, including novel designs, intelligent responses, high performance, and future applications. Novel designs provide crucial guidance for the intelligent responses of MICs, jointly influencing their performance. High performance of MICs promotes their applications in diverse emerging fields. Future applications of MICs will also in turn optimize the designs of MICs.

In this work, we systematically present the latest research results on MICs based on various biomimetic systems. Firstly, natural organisms with external stimulus‐driven behaviors, including snakes, hagfish, earthworms, mussels, and polar bears, are exhibited and discussed in detail. A special focus on the internal mechanisms and application perspectives is also given. Then, various shell, core, and coating materials used for preparing MICs are shown and listed, and some suggestions for the material selection of specific scenarios are also discussed. After that, various types of stimulus‐responsive microcapsules and coatings are introduced, which can be controlled by various external stimuli, including light, temperature, magnetic, mechanical, pH, and ions. The feasibility of stimulus‐responsive microcapsules and coatings in the design and development of MICs is demonstrated. Finally, we focus on the applications and development of such MICs, including anti‐corrosion, anti‐fouling, self‐lubricating, and temperature regulation. Although the research on MICs has made great progress, there is still great room for improvement. An important area of future work is to advance the practical utility supported by the fabrication of MICs for multi‐functional applications. In this regard, we believe that future research will aim to endow the materials with more intelligent responsiveness, improve the durability of materials, and further systematize the functions. Perspectives in detail are proposed as shown in **Figure** **10** and below.

### 6.1 Novel Designs

Firstly, to improve the stability, durability, and versatility of MICs, it is important to develop and select intelligent and adaptable materials with new structures for preparing MICs. Then, advances in fabrication techniques need to be developed for precisely controlling the size, shape, and morphology of microcapsules and also MICs. Fine‐tuning these physical properties and structures of MICs can significantly improve their stability, responsiveness, and compatibility, thereby broadening their functional efficacy. In addition, scalability challenges such as complex manufacturing processes, cost‐benefit balance, uniformity, and repeatability issues need to be solved when MICs are being brought into the industrialization process. Therefore, new techniques such as continuous flow microfluidic technology or high‐throughput electrospinning process could be used to realize the large‐scale and controllable synthesis of microcapsules. Meanwhile, the coating film formation process and performance evolution can be dynamically simulated by building a virtual production system using digital twin technology. This enables the optimization of process parameters (such as spray pressure and curing temperature), thereby enhancing the batch stability to the industrial standard. Furthermore, a comprehensive understanding of the intricate relationships between molecular structures and performance is essential for the development of high‐performance MICs. Therefore, advanced techniques such as diverse in situ characterizations which can significantly support the study of the structure‐activity relationship of materials, should be used. Finally, it is necessary to integrate machine learning algorithms (ML) and AI into the research of MICs to simplify the overly complex reaction process and reduce the cost of trial and error. ML and AI could also be applied to analyze large numbers of material databases and experimental data to predict the structure, properties, and stability of novel MICs. For example, the cracking behavior of microcapsules in MICs had been predicted by a combined finite element method and ML. After training 1031 datasets from finite element simulations using an artificial neural network, microcapsule cracking and healing agents releasing can be judged accurately and instantly without extensive computation.^[^ ^183^ ^]^ With the development of more experimental and theoretical databases, the novel designs of MICs are becoming more efficient, which is expected to lead to the development of MICs with desirable properties.

### 6.2 Intelligent Responses

The stimulus response of MICs has a direct impact on their properties. Various stimulus‐responsive types, such as temperature, light, pH, and ion have been integrated into one MIC, which exhibits attractive potential in industrial applications. However, the development and application of MICs are still hindered due to some technical limitations, such as slower response speed, limited stimulation response methods, and difficulties in real‐time monitoring. Firstly, although MICs can successfully respond in special operating conditions, these features are often not timely and accurate. To address this drawback, analyzing the response characteristics and mechanisms of MICs is an effective way to promote response sensitivity. Secondly, many single‐stimulus responses have different and insurmountable disadvantages, such as limitations of material selection for mechanical response, environmental pollution for pH response, and weak sensitivity for ion response. With the continuous advances of technology, the combination of different stimulus‐response strategies into multiple stimulus‐response strategies makes MICs more intelligent and accurate. For example, by imitating the ion channel mechanism of biological cell membranes, develop intelligent switch MICs that respond synergistically to pH, temperature, and mechanical force. Based on the octopus skin structure, develop intelligent MICs with multi‐field coupling responses of light, heat, and electricity. To achieve the real‐time monitoring of MICs, fluorescent or raman‐labeled molecules can be used to visualize the warning of corrosion or wear conditions within the MICs. Wireless sensor nodes (such as radio frequency identification devices) could also be embedded in the MICs to build a cloud‐based monitoring platform, enabling remote tracking of the operational status. Finally, irreversible damage to substrates may be caused by the stimulus when acting on MICs. Therefore, it is important to rationally design MICs with remote control performance and reduce their material damage.

### 6.3 High Performance

Firstly, the durability of MICs is a critical concern in actual applications. Among the many influencing factors on the durability of MICs, the bonding and stability between microcapsules and coatings in MICs are important for extending the service life of materials, which can be enhanced through surface treatment, interface modification, and post‐treatment. In addition, the preparation and treatment processes of MICs involve the use of harmful solvents. In the future, the selection of green materials and the production of environmentally friendly MICs may be a popular trend in the research of MICs. Finally, the low efficiency of MICs limits their application fields. Therefore, researchers need to optimize their multiscale structures, including molecular structures and nano/micro structures, to maximize their efficacy in a variety of applications. While improving the performance of MICs, it is necessary to pay attention to the environment aspects of the MICs, such as material safety certification, sustainability, biodegradability, regulatory compliance, and the lack of standardization. The sustainable development of future shell/core/coating materials can focus on intelligent environmental compliance systems, which can be full‐process ecological monitored from raw material traceability to waste disposal using blockchain and AI technologies. At the same time, unified biodegradability evaluation standard should be established to promote the large‐scale application of green alternative materials such as water‐based resins and bio‐based curing agents. Furthermore, a dedicated database for MICs, which integrates material toxicology data, could be established to promote the opening of the FDA pre‐review channel and significantly shorten the approval cycle for new environmentally friendly materials.

### 6.4 Potential Applications

MICs have exhibited significant promise in early applications in various fields such as anti‐corrosion, anti‐fouling, self‐lubricating, and temperature regulation. However, their potential applications in many real‐world scenarios remain largely untapped. For example, it is crucial for weapons and equipment to avoid radar, infrared, and laser detection in the military scenario. Stealth performance is expected to be achieved by incorporating absorbent materials into novel MICs for intelligent weapon and equipment protection. Secondly, self‐driving cars and additional safety devices on cars rely on lidar technology. This means that cars need to be kept clean, which can be solved by MICs with self‐cleaning functions. In addition, the main anti‐corrosion measures for seawater systems, such as circulating water systems and circulating water treatment systems in domestic nuclear power plants, are coating protection. From the anti‐corrosion mechanism of MICs, it can be seen that MICs have potential application value and broad market application prospects for seawater systems in nuclear power plants. Finally, the ability of MICs to actively respond to internal or external stimuli is also considered an effective way to create efficient orthopedic implant coatings, which may open a new avenue for future research and application of orthopedic implants. However, the application of MICs in the field of life and health still faces many obstacles related to standardization, such as protocol development challenges and implementation difficulties. For example, medical MICs must simultaneously meet both biocompatibility and stimulus‐responsive properties. The existing testing standards do not cover dynamic functional assessment. The traditional coating durability tests (such as salt spray experiments) cannot simulate the in vivo environment (pH/enzyme dynamic changes), resulting in a disconnection between the accelerated aging data and the actual service performance. In the future, a grading and standardization system can be established. Differentiated testing requirements can be set based on risk levels (contact duration/implant depth), for example, short‐term skin contact coatings can simplify the cytotoxicity testing process. Furthermore, a dedicated pre‐certification database for MICs (including material‐function‐toxicity correlation data), which is the key infrastructure for resolving the bottleneck of standardization in the health sector, could be established. This database can accelerate the innovation of regulatory science and promote interdisciplinary collaborative design.

In conclusion, MICs show considerable advantages in the treatment of anti‐corrosion, anti‐fouling, self‐lubricating, and temperature regulation. However, there are still challenges in improving their performance, such as durability and sensitivity. The combination of novel techniques, theoretical studies, and AI techniques will provide deeper insights into the basic research and future applications of MICs. This requires the joint efforts of multiple fields, including chemistry, materials, and computer science. We look forward to further exciting research advances in this area.

## Conflict of Interest

The authors declare no conflict of interest.
